# Direct and Indirect Interfacial Electron Transfer at a Plasmonic p-Cu_7_S_4_/CdS Heterojunction

**DOI:** 10.1021/acsnano.4c14556

**Published:** 2025-01-02

**Authors:** Zhicheng Yang, Nandan Ghorai, Shengxiang Wu, Sheng He, Tianquan Lian

**Affiliations:** Department of Chemistry, Emory University, Atlanta, Georgia 30322, United States

**Keywords:** nanorods, type-II heterostructure, plasmonic semiconductor, hot electron transfer, heat transfer, Cu_7_S_4_

## Abstract

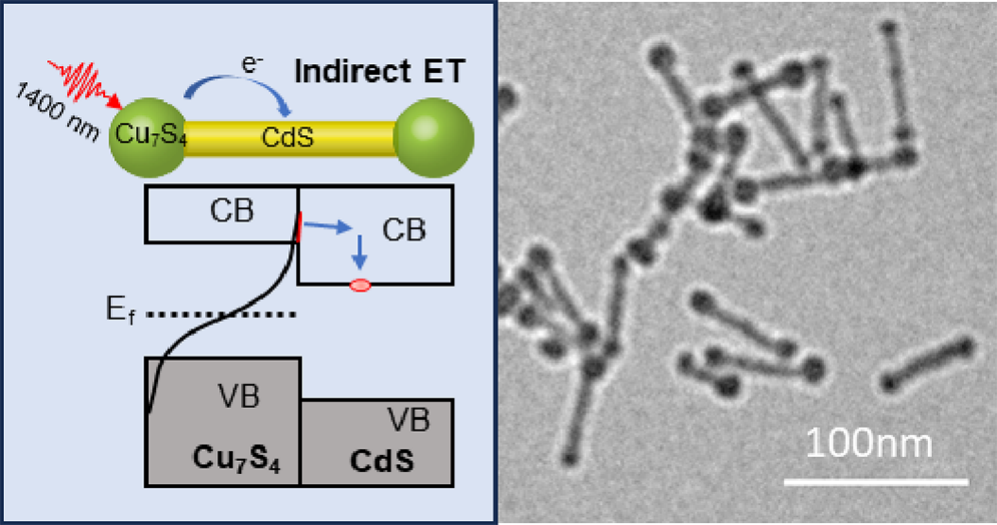

Plasmonic semiconductors exhibit significant potential for harvesting near-IR solar energy, although their mechanisms of plasmon-induced hot electron transfer (HET) are poorly understood. We report a transient absorption study of plasmon-induced HET in p-Cu_7_S_4_/CdS type II heterojunctions. Near-IR excitation of the p-Cu_7_S_4_ plasmon band at ∼1400 nm leads to ultrafast HET into the CdS conduction band with a time constant of <150 fs and a quantum efficiency of ∼0.054%. The injected hot electrons remain in CdS with an amplitude-weighted average lifetime of 1.9 ± 0.5 ns, significantly longer than that in Au/CdS heterostructures, suggesting that plasmonic semiconductors can slow down charge recombination due to the presence of a bandgap. The excited near-IR plasmon does not decay by coupling to the interfacial charge transfer transition, likely due to its energy mismatch. This study provides a detailed mechanistic understanding and possible directions for improving plasmonic HET in plasmonic semiconductor heterojunctions.

## Introduction

**Scheme 1:**
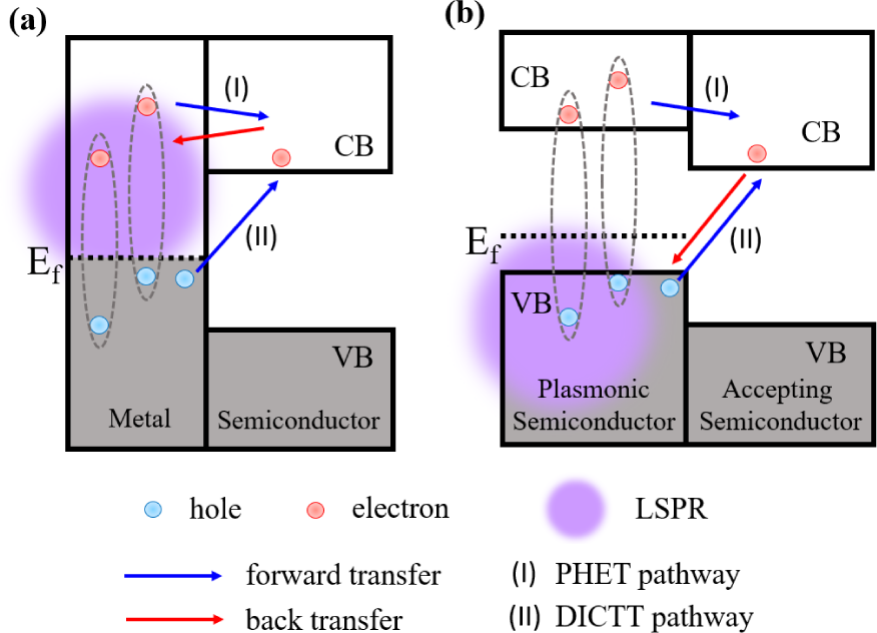
(a) In a Schottky Junction, the Transferred Electron Can Rapidly Transfer Back to Empty Bands in the Metal; (b) in a Heterojunction System, the Transferred Electron Can Only Recombine with the Hole in the VB of the Plasmonic Semiconductor

Plasmonic hot carriers provide tremendous opportunities for potential applications in photodetectors, photovoltaics, and photocatalysis.^1−3^ After photoexcitation, the confined collective oscillation of free carriers, also termed localized surface plasmon resonance (LSPR), nonradiatively decays to generate hot carriers (nonthermalized) through bulk (Drude-like)^4^ or surface (Landau-damping)^5^ scattering on the 10 fs time scale; these hot carriers can be subsequently extracted into adjacent semiconductors or molecules (Scheme 1) to drive photochemistry.^1,6−11^ This process is often thought to follow the indirect plasmon-induced hot electron transfer (PHET) pathway, in which the transfer of hot carriers generated within the plasmonic particle competes with rapid hot electron thermalization (<100 fs) through electron–electron scattering (e–e) followed by electron–phonon scattering (e–ph) (∼a few ps).^2,9^ To achieve high hot electron transfer (HET) efficiency, interfacial charge transfer is required to be faster than the ultrafast cooling process.^1,6,12−15^ Thus, the quantum efficiency (QE) for this indirect PHET process is usually low.^6,16^ Furthermore, the transferred hot electron in the semiconductor or molecular acceptor can quickly back-transfer to the large and continuous density of empty states in the metal at energy below the semiconductor conduction band or the molecular LUMO, further reducing the efficiency of utilizing the hot carriers under steady-state conditions (Scheme 1a).

Significant efforts have been devoted to improving the efficiency of hot electron transfer. It has been reported that indirect PHET QEs can be improved by reducing the metal nanoparticle size in Au-tipped CdS nanorods and Ag/TiO_2_ by increasing the surface damping contribution to plasmon decay.^17,18^ Previously, some of us reported plasmon-induced interfacial charge transfer transition (PICTT) in Au-tipped CdSe nanorods (NRs) with a high QE (>24%).^19^ Mixing of the semiconductor (or adsorbate) and metal orbitals at the interface leads to the formation of a direct interfacial charge transfer transition (DICTT), and plasmons can decay by exciting these transitions, directly promoting electrons from metallic nanocrystals (NCs) to the adjacent semiconductor or chemisorbed molecules.^19−21^ In this direct PICTT pathway, competition with hot electron relaxation is avoided; hence, a higher QE can be achieved. Recently, the PICTT pathway has also been observed in Ag-sensitized TiO_2_ nanoporous films,^18^ epitaxial Ag/CdS icosapods,^22^ and Au/TiO_2_ core–shell NRs.^23^

In addition to increasing the HET QE, another major challenge in the utilization of plasmonic hot carriers is the rapid back electron transfer to empty band states in the metal, often on the few ps time scale.^16−19^ Because of the slow rates of typical chemical reaction kinetics (microseconds to seconds),^1,24−26^ it is necessary to elongate the extracted electrons’ lifetime by slowing down back electron transfer. One possible way to slow back electron transfer is to use heavily doped semiconductors with a plasmon band. Recently, a class of self-doped plasmonic semiconductors has been developed (e.g., n-WO_3–*x*_, n-MoO_3–*x*_, p-Cu_2–*x*_S, p-Cu_2–*x*_Se).^3,27,28^ In the case of well-developed Cu_2–*x*_E (E = S, Se; 0 < *x* ≤ 1), the plasmon band in the near-IR region originates from the large free carrier concentrations caused by the Cu^+^ vacancy and can be adjusted by changing the size, geometry, Cu stoichiometry,^28−32^ crystal structure,^33,34^ and dielectric properties of the surrounding environment. Besides being more cost-effective than noble metals and versatile for controlling LSPR, the semiconductor nature of these self-doped plasmonic materials offers opportunities to develop novel plasmonic heterojunctions by interfacial engineering.^6,7,35−37^ In a plasmonic semiconductor/semiconductor heterojunction, such as Cu_2–*x*_Se/CdSe, transferred electrons in the accepting semiconductor CB can recombine only with the VB holes in the plasmonic semiconductor due to the presence of a bandgap in a plasmonic semiconductor (Scheme 1b), and the large excess energy is expected to slow down the back electron transfer rate. This notion is supported by a recent report of ∼130 ps back electron transfer time in Cu_2–*x*_Se/CdSe NCs,^6^ which is considerably longer than that of Au/CdSe and Au/CdS NRs.^17,19^ Even a longer lifetime of transferred hot electrons in plasmonic Cu_7_S_4_/CdS nanoplates (NPLs) has also been reported.^7^

In this work, we study plasmon-induced hot electron transfer in p-Cu_7_S_4_/CdS barbell heterostructures, with Cu_7_S_4_ NCs acting as p-doped plasmonic semiconductor materials and CdS (NRs) acting as the accepting semiconductor. Using transient absorption spectroscopy, we show that the excitation of the plasmon band at 1400 nm leads to hot electron transfer from Cu_7_S_4_ to the CdS domain and heating of the nanorod. We also investigate the electron transfer process resulting from a direct excitation of Cu_7_S_4_ to the CdS domain interfacial charge transfer transition at ∼600 nm.

## Results and Discussion

### Structural Characterization

**Figure 1:**
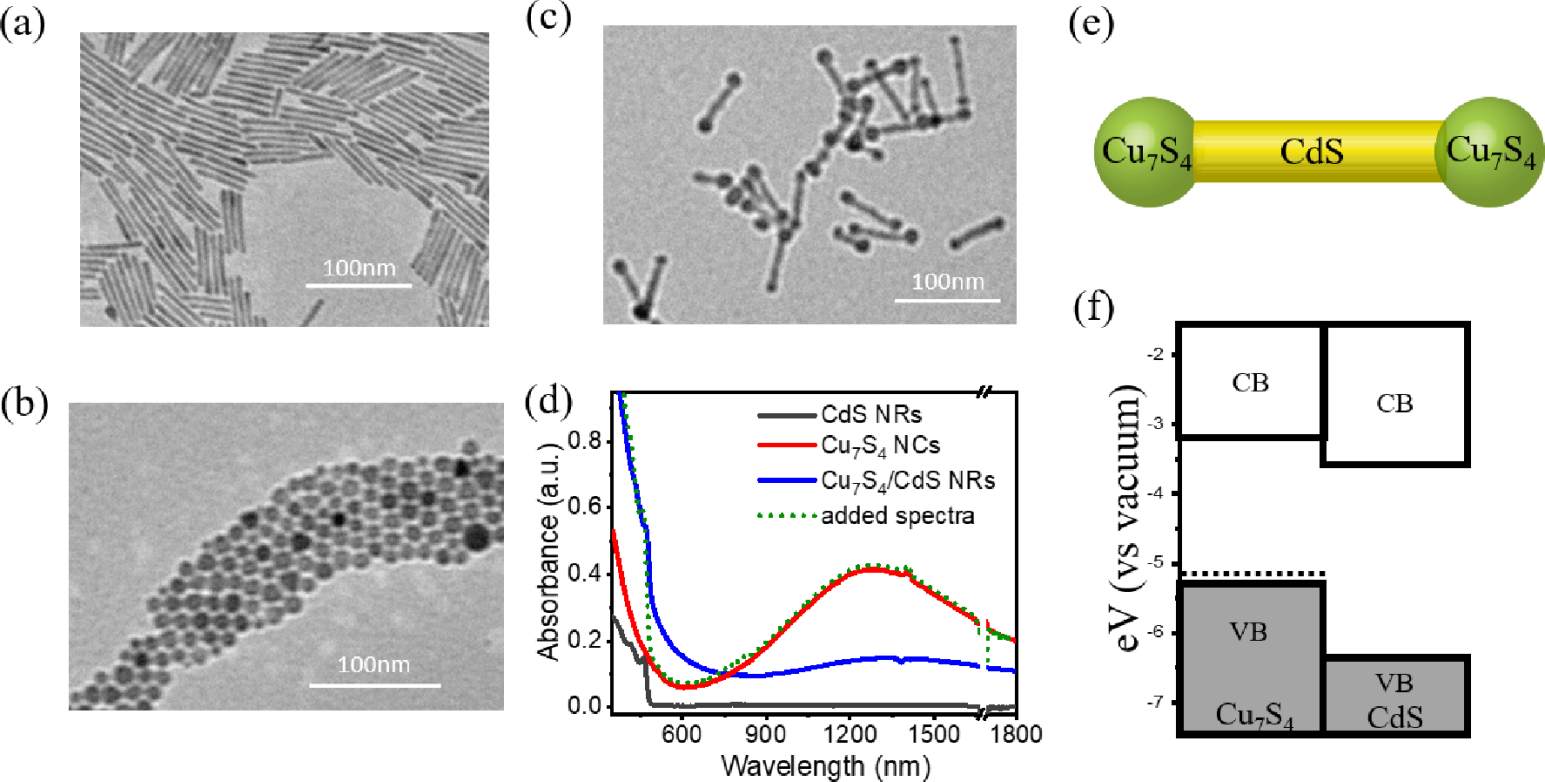
Transmission electron microscopy (TEM) images of (a) CdS NRs, (b) Cu_7_S_4_ NCs, and (c) Cu_7_S_4_/CdS NRs. Inset scale bar: 100 nm. (d) UV–vis-NIR absorption spectra of CdS NRs, Cu_7_S_4_ NCs, and Cu_7_S_4_/CdS NRs (colored solid lines). Also shown is the fit to the Cu_7_S_4_/CdS spectra (green dotted line) by using a linear combination of the absorption spectra of bare CdS NRs (solid gray line) and bare Cu_7_S_4_ NCs (solid red line). The deposition of Cu_7_S_4_ greatly changes the electronic structure of CdS. (e) Schematic representation of Cu_7_S_4_/CdS NRs. (f) Energy alignment of the Cu_7_S_4_/CdS heterostructure.

The Cu_7_S_4_/CdS heterostructure used in the present study is prepared by a heat decomposition method, the details of which are provided in Note S1. We first prepared CdS NRs through a well-developed seeded-growth method,^38^ then decorated both tips with Cu_7_S_4_ NCs. The TEM image of CdS NRs (Figure 1a) shows a uniform 1D morphology with an average length and diameter of 53.89 ± 0.58 nm and 4.32 ± 0.06 nm, respectively, and the length and diameter distributions are shown in Figure S1a–c. The growth of Cu_7_S_4_ NCs on the tips of the CdS NRs is confirmed by TEM images shown in Figures 1c and S1d. Analysis of the TEM images shows that the percentages of NRs with zero, one, or two tips are 6.4%, 9.5%, and 84.1%, respectively, as shown in Figure S1e. Thus, most Cu_7_S_4_/CdS heterostructures have Cu_7_S_4_ tips and can be represented by a barbell-like structure shown in Figure 1e. The average size of the Cu_7_S_4_ domain is determined to be 12.20 ± 0.31 nm (Figure S1f). As a control sample, we have also prepared bare Cu_7_S_4_ NCs with a similar diameter (11.40 ± 0.37 nm) (Figures 1b and S1h).

The UV–vis-near-IR absorption spectra of CdS NRs, Cu_7_S_4_ NCs, and Cu_7_S_4_/CdS NRs are shown in Figure 1d. CdS nanorods show a distinct band edge exciton transition at 463 nm and Cu_7_S_4_ NCs show a clear near-IR plasmon band centered at 1265 nm and a higher energy band at <600 nm that is attributable to interband transition in Cu_7_S_4_, consistent with the reported bandgap of Cu_2–*x*_S.^39^ The spectra of Cu_7_S_4_/CdS NRs show the Cu_7_S_4_ near-IR plasmon band, the CdS band-edge absorption peak at ∼470 nm, and a new absorption feature at 500 to 800 nm. This can be clearly seen by a comparison of the Cu_7_S_4_/CdS NR spectrum and the sum of the absorption spectra of Cu_7_S_4_ and CdS NRs (green line in Figure 1d). We attribute this new feature to the interfacial charge transfer transition between the Cu_7_S_4_ and CdS domains due to the mixing of their electronic levels at the interface. Similar charge transfer transitions have been observed in other semiconductor heterostructures.^40−44^ Compared to Cu_7_S_4_ NCs, the near-IR plasmon band of Cu_7_S_4_ in the Cu_7_S_4_/CdS heterostructure is red-shifted from 1265 to 1343 nm (Figure 1d), which may be caused by the change of dopant density, size, shape, or dielectric environment.

To examine the origin of the change in the near-IR plasmon band, we prepared another set of Cu_7_S_4_ NCs and Cu_7_S_4_/CdS NCs in which the Cu_7_S_4_ doping levels are controlled by varying the oxygen exposure time in the synthesis process,^31^ as shown in Figure S2a. This result shows that the difference in plasmon bands in Cu_7_S_4_ NCs and Cu_7_S_4_/CdS NCs can be attributed mainly to different doping levels in the Cu_7_S_4_ domain. It has been reported that plasmons could be strongly damped by coupling to the interfacial charge transfer transition of a plasmonic heterostructure through the PICTT mechanism.^19,22^ However, this phenomenon is not observed in the Cu_7_S_4_/CdS NRs studied in the current work. The similar plasmon bands in this new set of Cu_7_S_4_ NCs and Cu_7_S_4_/CdS NCs also enable the subtraction of the Cu_7_S_4_ contribution from the total absorption of the Cu_7_S_4_/CdS NC, as shown in Figure S2b. Fitting of the subtracted spectrum reveals clearly the interfacial charge transfer transition with an onset energy of ∼1.5 eV. Moreover, according to the estimated energy levels (shown in Note S2), the Cu_7_S_4_/CdS heterojunction shows a type II energy alignment (Figure 1f), consistent with previous reports.^7,36,37^

### Plasmon-Induced Electron and Heat Transfer in Cu_7_S_4_/CdS Heterojunctions

**Figure 2:**
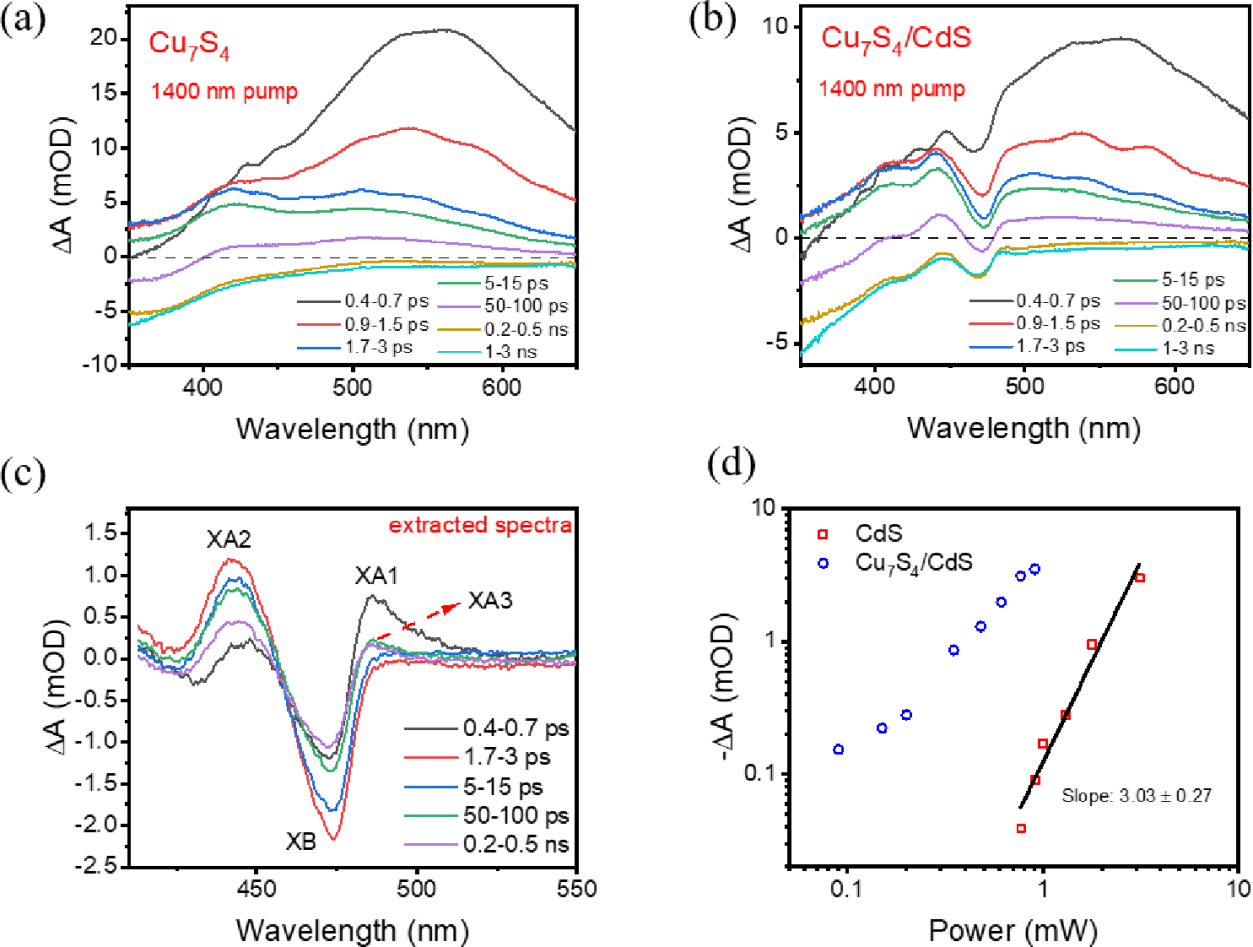
Transient absorption spectra (TAs) of (a) Cu_7_S_4_ NCs and (b) Cu_7_S_4_/CdS NRs after 1400 nm excitation (1.33 mJ/cm^2^). (c) The extracted spectra of the CdS domain in Cu_7_S_4_/CdS NRs obtained by subtracting the scaled Cu_7_S_4_ spectra in panel (a) from the Cu_7_S_4_/CdS spectra in panel (b). (d) The dependence of the extracted XB signal amplitude at 473 nm as a function of excitation power at 1400 nm, representing the extent of hot electron transfer from Cu_7_S_4_ to CdS in Cu_7_S_4_/CdS NRs. Also shown for comparison are the signals in CdS NR-only samples, showing a much smaller signal size and different power dependence.

**Figure 3:**
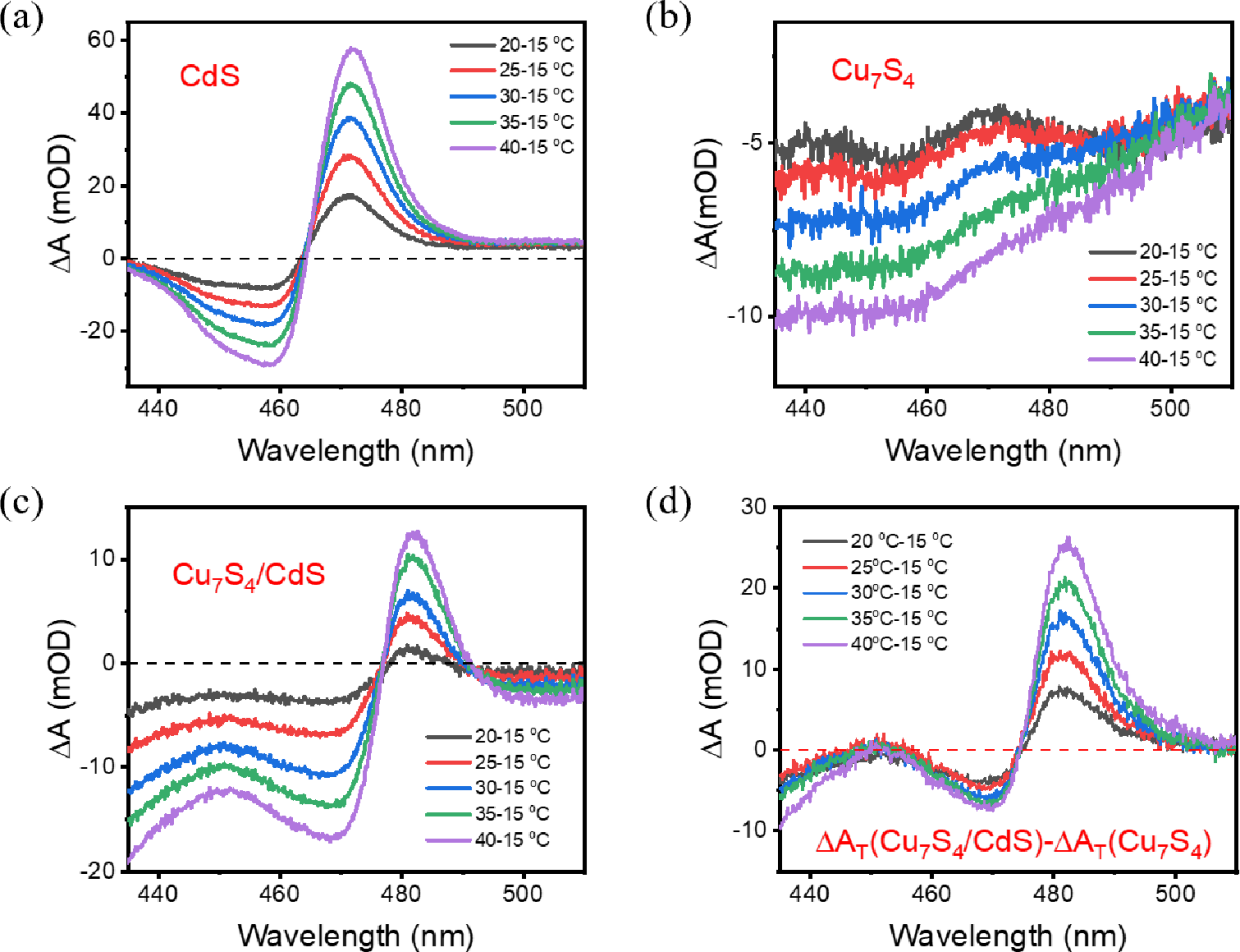
Heat-induced difference absorption spectra of (a) CdS NRs, (b) Cu_7_S_4_ NCs, and (c) Cu_7_S_4_/CdS NRs calculated by using the absorption spectra at 15 °C subtracted from those at higher temperatures. (d) Extracted heat-induced difference absorption spectra of the CdS domain in Cu_7_S_4_/CdS NRs calculated by subtracting the spectra in (b) from those in (c). The difference absorption spectra of Cu_7_S_4_ NCs are scaled for ease of subtraction.

We employed transient absorption (TA) spectroscopy to investigate the excited state dynamics of Cu_7_S_4_ NCs and Cu_7_S_4_/CdS NRs. Figure 2a shows the TA spectra of Cu_7_S_4_ at the indicated delay times after the photoexcitation of the plasmon band at 1400 nm. At short delay times, a broad absorption feature centered at ∼550 nm appears instantaneously, as shown in the average spectrum at a delay time of 0.4–0.7 ps, which decreases in amplitude and shifts to a shorter wavelength on the picosecond time scale. This spectral signature is consistent with the broadening of the LSPR band upon photoexcitation.^36,45,46^ Interestingly, beginning at 0.2 ns, a long-lived ground state bleach (GSB) signal is observed at a wavelength shorter than 500 nm, similar to the difference spectra from directly heated Cu_7_S_4_ NCs (Figure 3b). Thus, we attribute the GSB to the increased lattice temperature resulting from the plasmon-induced heating of the Cu_7_S_4_ NC.^6,15^ Burda et al. reported similar spectral features at 550 nm upon excitation of Cu_1.8_S NCs by a visible pump and attributed this feature to trapped carrier-induced absorption in the Cu_1.8_S NCs.^47^ A similar feature was observed in the present system, and a contribution of carrier trapping processes to the early time spectra cannot be excluded.

For Cu_7_S_4_/CdS NCs, in addition to the spectral features in the Cu_7_S_4_ domain discussed above, a negative feature (bleach) at ∼473 nm can be observed (Figure 2b). This corresponds to the decrease of the CdS excitonic absorption caused by the conduction band edge electron-induced state filling effect, which confirms electron injection from Cu_7_S_4_ to the CdS domain in the heterostructure. The extracted bleach amplitudes at 473 nm (Figure 2d) reveal a superlinear dependence on the excitation fluences. Under the same excitation fluence, a negligible signal is observed for bare CdS NRs (Figure S3). At much higher fluence, the CdS NRs show a bleach of the exciton band that exhibits a cubic dependence on the fluence (Figure 2d), indicating that the signal is caused by three-photon absorption. The comparison of CdS and Cu_7_S_4_/CdS NCs suggests that the bleach feature for Cu_7_S_4_/CdS NCs is not caused by multiphoton absorption of the CdS NRs.

In order to obtain the pure spectral features corresponding to the CdS domain, we subtract the contribution of Cu_7_S_4_ from the spectrum of Cu_7_S_4_/CdS. To do this, we first compare the TA spectra (Figure S4a) and kinetics (Figure S4b) of Cu_7_S_4_ and Cu_7_S_4_/CdS. The comparison shows that the spectra of Cu_7_S_4_/CdS match well with the scaled spectra of bare Cu_7_S_4_ except for the region around 473 nm, in which an additional derivative-like feature is observed in Cu_7_S_4_/CdS. Similarly, the kinetics of Cu_7_S_4_/CdS match well with the scaled kinetics of Cu_7_S_4_ at 425, 500, and 550 nm, and the only mismatch is observed at 473 nm. The scaling of the signal amplitude of the bare Cu_7_S_4_ sample is needed in the comparison because of its lower optical density at the excitation wavelength (1400 nm). These comparisons confirm that the TA spectra of Cu_7_S_4_/CdS NRs can be considered a sum of the Cu_7_S_4_ and CdS domains. The contribution of the Cu_7_S_4_ signal can be subtracted by comparing it with the TA spectra of bare Cu_7_S_4_. The subtracted TA spectra of the CdS domain upon 1400 nm photoexcitation are shown in Figure 2c. As will be discussed further, these features contain the contribution of plasmon-induced hot electron and heat transfer from the Cu_7_S_4_ to CdS domains.

To investigate the effect of heating on the TA spectra of Cu_7_S_4_/CdS NRs, we measured the temperature-dependent absorption spectra of bare Cu_7_S_4_ and bare CdS separately. For bare CdS NRs, a heating-induced red-shift of the absorption spectra (Varshni effect)^48^ is observed (Figure S5a), thus resulting in a derivative feature at the band edge in the difference spectra (Figure 3a). For bare Cu_7_S_4_ NCs, with the increase in temperature, the absorption intensity is reduced in the probe range, causing a broad bleach signal (Figure 3b). The spectral features from both of these components exist in Cu_7_S_4_/CdS NRs (Figure 3c). Moreover, the heating-induced signal amplitude is linearly dependent on temperature differences (Figure S6a–c). To obtain the derivative feature in the CdS domain, the broad bleach signal of the Cu_7_S_4_ domain is subtracted from the spectra of Cu_7_S_4_/CdS NRs (Figure S7). As shown in Figure 3d, the shape of the extracted spectra is similar to that of the bare CdS NRs. The amplitude also follows the linear relationship with temperature change (Figure S6d) and can be used to probe the temperature change in the CdS domain. Moreover, the extracted spectra are red-shifted, and the ratio of positive absorption and the bleach signal is increased, which may be attributed to the perturbed electronic structure in the CdS domain in the heterostructure.

**Figure 4:**
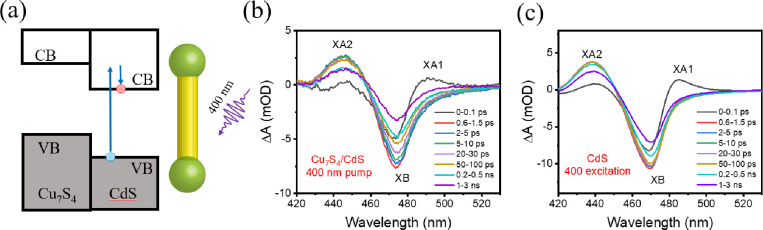
(a) Schematic of the generation of the CdS band edge exciton. TA spectra of Cu_7_S_4_/CdS NRs (b) and bare CdS NRs (c) at the indicated delay time windows after 400 nm photoexcitation.

To understand the spectral signature of electron transfer to the CdS domain, we measured the TA spectra of Cu_7_S_4_/CdS NRs under 400 nm excitation. For this measurement, the excitation fluence was adjusted to ensure that the average absorbed photons per Cu_7_S_4_/CdS nanobarbell is ≪1. As shown in Figure 4b, the TA spectra of Cu_7_S_4_/CdS NRs display a red-shifted absorption feature (XA1) at early delay times, attributed to the red-shifted band edge exciton transition caused by hot electrons in CdS NRs.^49^ The XA1 feature decays rapidly as hot electrons cool on the 100 fs time scale, forming a bleach of the first excitonic band in CdS (XB), which has been shown to be caused by the state filling effect of electrons at the conduction band edge.^49^ The positive feature at higher energy than the band edge exciton (XA2) is attributed to the shifting of higher energy transitions in CdS caused by the photogenerated exciton.^49^ Similar spectral features are observed for bare CdS NRs (Figure 4c), while no signal is observed from bare Cu_7_S_4_ under the same conditions (Figure S8). Moreover, the spectra of Cu_7_S_4_/CdS NRs can overlap very well with those of CdS NRs after accounting for their slight difference in exciton band positions (Figure S9). Hence, the TA spectra of Cu_7_S_4_/CdS NRs with 400 nm excitation are caused by the presence of conduction band edge electrons in the CdS domain.

**Figure 5:**
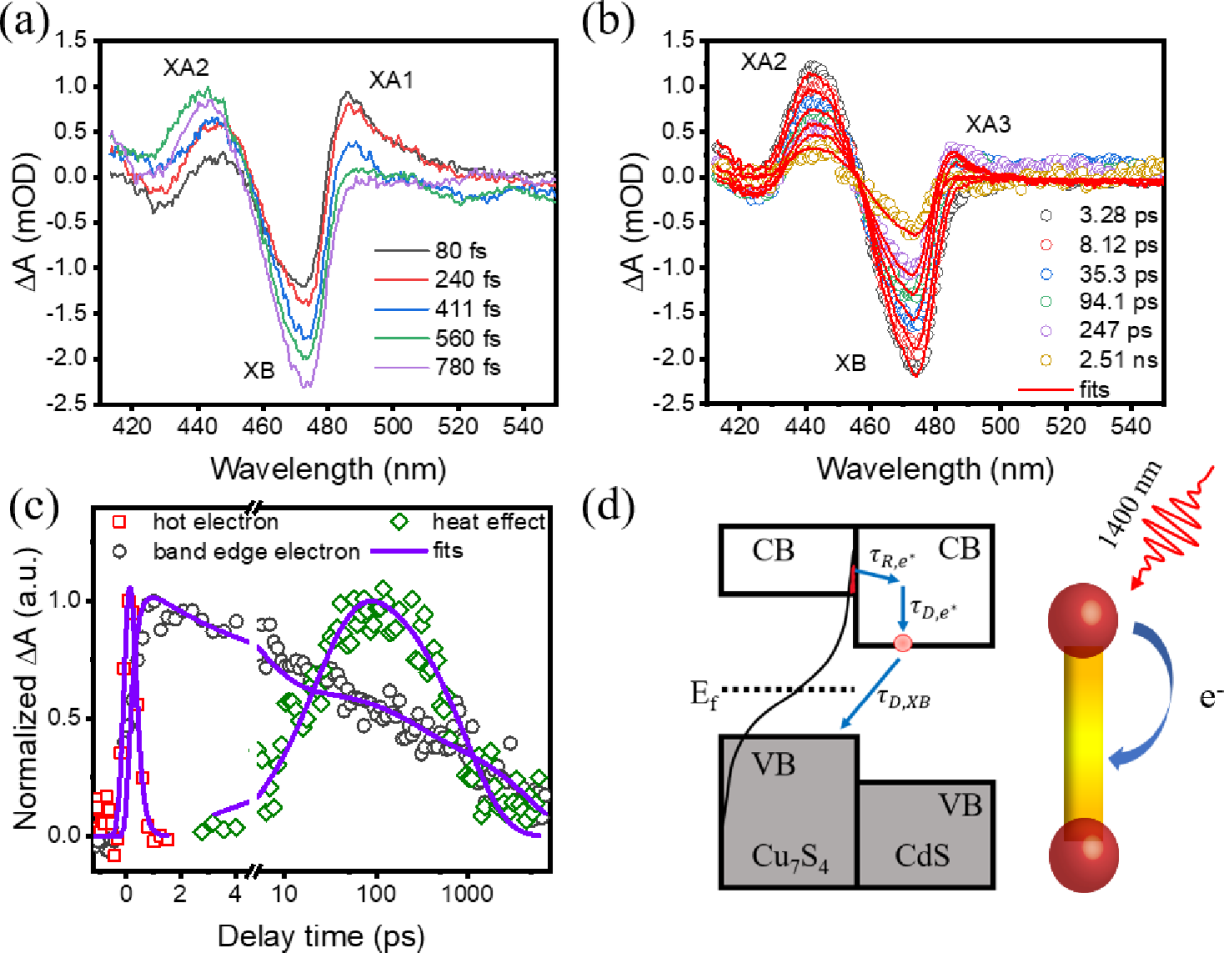
(a) Extracted spectra at early delay times showing the hot electron feature (XA1) and band edge electrons (XB and XA2). (b) Extracted spectra at later delay times showing band edge electrons (XB and XA2) and the heat-induced signal (XA3). A two-component fitting is applied. (c) Red open squares: kinetics for transferred hot electrons; black open circles: kinetics for band edge electrons; green open diamonds: kinetics for heat-induced signals; purple solid lines: fitting results according to the equations in the main text. The kinetics of hot electrons and band edge electrons are measured and normalized from the extracted spectra at 485 and 474 nm, respectively. The kinetics for the heat effect is obtained from scaled β(*t*). (d) Schematic of plasmon-induced hot electron transfer and a heat transfer process. In the right panel, the red tip represents the heated Cu_7_S_4_ domain, while the orange part and the yellow part represent the heated and unheated parts in CdS NRs, respectively.

With the separately obtained spectra for the heat effect and CdS conduction band edge electrons, the evolution of the extracted CdS spectra of Cu_7_S_4_/CdS NRs (Figure 2c) can be fully understood. At early delay times (Figure 5a), the plasmon-induced hot electron rapidly transfers to CdS, giving rise to the XA1 feature, which cools to the band edge and generates the XB feature. At later delay times (Figure 5b), the heating-induced feature (XA3) forms and then disappears, which can be attributed to heat transfer from Cu_7_S_4_ to CdS and subsequent dissipation into the surroundings.

The kinetics for transferred hot electrons and band edge electrons can be obtained through XA1 at 485 nm and XB at 474 nm, respectively. Note that the heating-induced signal has a negligible contribution at 474 nm (Figure 3d), making it suitable for representing the band edge electrons. Additionally, a two-component global fitting is performed to determine the time-dependent contribution of the heating-induced signal after 2 ps, by when the hot electrons have completely relaxed. The spectra after 2 ps are fitted to a linear combination of the spectral changes caused by the band edge electrons (Δ*A*_e_(λ)) and heat transfer (Δ*A*_heat_(λ)), as described in eq 1.
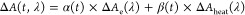
1

Herein, α(*t*) and β(*t*) represent the amplitudes of band edge electron and heat transfer spectral components, respectively. All heat-induced spectra (Figure 3d) and band edge electron-induced spectra (Figure 4b) can separately overlap very well after scaling (Figures S10 and S9b). Therefore, we selected the spectra from 40 to 15 °C in Figure 3d and the spectra from 0.6 to 1.5 ps in Figure 4b to represent the heat transfer and electron transfer in our fitting analysis, respectively. The spectra at later delay times are well-fitted by eq 1, as shown in Figures 5b, S11, and S12. Hence, the heat transfer process that occurs can be well described by β(*t*) obtained from the fit. Moreover, the contribution of band edge electrons (>2 ps) agrees well with the kinetics collected at 474 nm (Figure S13).

The injected hot electron kinetics at 485 nm (Figure 5c) can be fitted with a single exponential rise and single decay function convoluted with the IRF according to eq 2,
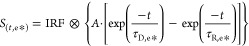
2

The hot electron rising time constant (τ_R,e*_) is faster than our instrument response time ∼150 fs, and the hot electron decay time constant (τ_D,e*_) is determined to be 0.19 ± 0.07 ps.

The kinetics of XB can be fitted with a single exponential rise and triexponential decay function convoluted with the IRF (eq 3).
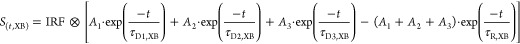
3

As shown in Figure 5c, the measured XB kinetics can be well fitted by eq 3, and the fitting parameters are listed in Table 1. According to the fit, the XB rising constant (τ_R,XB_) is 0.23 ± 0.04 ps, which is close to the hot electron decay constant of 0.19 ± 0.07 ps, reflecting the cooling process for the injected hot electron to the conduction band edge, as indicated in Figure 5d. The XB decay is well described by a three-exponential decay with the decay time constants of (τ_D*i*,XB_, *i* = 1–3 of 5.0 ± 1.3 ps, 249 ± 145.0 ps, and 4.7 ± 1.1 ns. This XB decay is attributed to back electron transfer from the CdS CB to Cu_7_S_4_ VB and trapping of the electron by defect states.^50^ Interestingly, the amplitude-weighted average lifetime of the transferred electron is 1.9 ± 0.5 ns, which is longer than the previously reported lifetime of ∼130 ps in Cu2_–*x*_Se/CdSe NRs.^6^ Both these lifetimes in plasmonic semiconductor/semiconductor junctions are much longer than the ∼1.5 ps lifetime of transferred electrons in plasmonic metal/semiconductor junctions of Au/CdS and Au/CdSe nanorods.^17,19^ We attribute the longer lived charge separated states in the former to the presence of the band gap in the plasmonic semiconductor, for which back electron transfer is slowed by the need to dissipate much larger excess energy, as shown in Figure 5d.

**Table 1:** Fitting Parameters for the Band Edge Electrons of Cu_7_S_4_/CdS Heterostructures after 1400 and 600 nm Excitation According to Eq 3

Excitation Wavelength (nm)	τ_R,XB_ (ps)	τ_D1,XB_ (ps)	τ_D2,XB_ (ps)	τ_D3,XB_ (ns)	*A*_1_	*A*_2_	*A*_3_
1400 (PHET)	0.23 ± 0.04	5.0 ± 1.3	249 ± 145.0	4.7 ± 1.1	43. 8% ± 4.1%	17.9% ± 3.9%	38.3% ± 3.8%
600 (DICTT)	IRF limited	5.3 ± 1.1	102 ± 17.4	5.6 ± 1.1	31.3% ± 2.2%	30.4% ± 2.2%	38.3% ± 1.9%

For the heat or vibrational energy transfer (VT) process, the kinetics could be fitted by a single exponential rise and a single exponential decay function, according to eq 4.
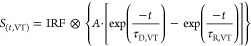
4

In eq 4, the phonon–phonon scattering raises the lattice temperature with a time constant τ_R,VT_, and heat dissipation to the surrounding cools down the NRs with a time constant of τ_D,VT_. According to the fitting result shown in Figure 5c, the heating and cooling time constants are 22.6 ± 2.6 and 972.7 ± 87.2 ps, respectively. Moreover, the CdS domain reaches the highest lattice temperature increase of 0.38 K at ∼92 ps (shown in Note S4).

From the amplitude of the CdS exciton bleach, we can also estimate the QE of plasmon-induced hot electron transfer with 1400 nm excitation. The details of the calculation are shown in Note S3. Under our experimental conditions, the HET QE can be estimated to be ∼0.054%. This result is similar to the previous report of the HET QE from Cu_2–*x*_Se to CdSe in Cu_2–*x*_Se/CdSe heterostructures,^6^ in which hot electron transfer occurs through a thermionic photoemission process. We speculate that a similar mechanism is responsible for HET in Cu_7_S_4_/CdS. This hypothesis is supported by the high nonlinear dependence of the HET signal on the excitation fluence shown in Figure 2d. Because of the large band gap in Cu_7_S_4_ (∼2.14 eV), it requires the absorption of multiple near-IR photons to reach a high enough electronic temperature to generate an electron population in the Cu_7_S_4_ conduction band. For this reason, the HET QE for this pathway is low. The HET QE is significantly lower than that in the Au-tipped CdS NRs (∼1–18%),^16,17^ Au/TiO_2_,^23^ or Ag/TiO_2_,^18^ in which one absorbed photon can generate hot electrons with sufficient energy to overcome the lower Schottky barrier between the metal Fermi level and the semiconductor conduction band. Thus, in comparison with plasmonic metal NPs, the band gap in the plasmonic semiconductor can lengthen the lifetime of transferred hot carriers but also lowers the HET QE.

### Direct Interfacial Charge-Transfer Transition (DICTT) in the Cu_7_S_4_/CdS Heterojunction

**Figure 6:**
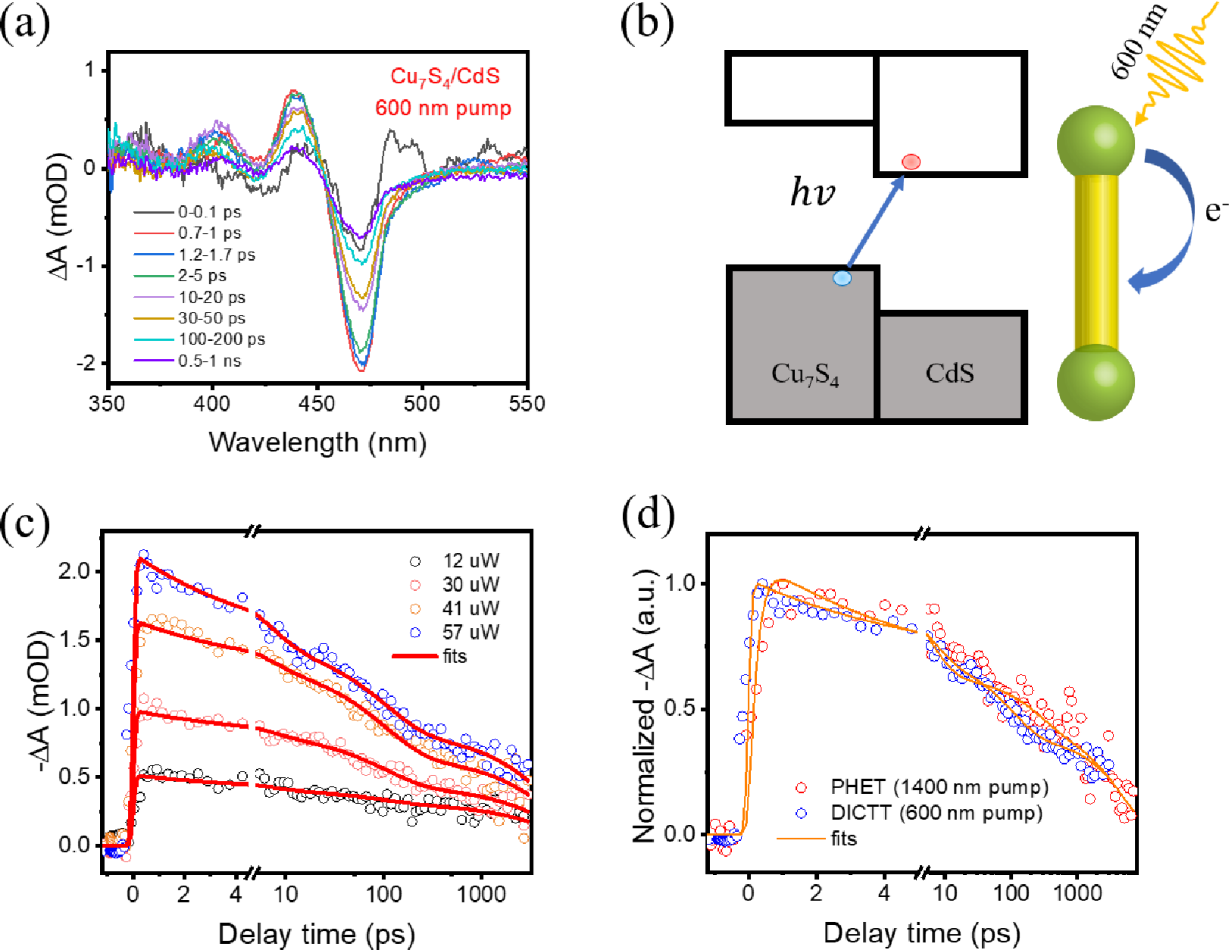
Transient absorption spectra and kinetics measured with 600 nm photoexcitation of DICTT. (a) TA spectra of Cu_7_S_4_/CdS NRs at indicated delay times, (b) schematic of the DICTT process in Cu_7_S_4_/CdS NRs, (c) global fitting for XB kinetics at 473 nm measured with various excitation powers. (d) Comparison of XB kinetics for CdS band edge electrons measured at excitation wavelengths of 1400 nm (red open circles) and 600 nm (blue open circles).

The absorption spectrum of Cu_7_S_4_/CdS NRs (Figures 1 and S2) also shows a new absorption feature at ∼500–800 nm that is attributed to a direct interfacial charge transfer transition (DICTT) from the Cu_7_S_4_ valence band to the CdS conduction band. We also measured the transient absorption spectra with excitation of this DICTT band at 600 and 800 nm. The TA spectra with 600 nm excitation (Figure 6a) show pronounced XB at the lowest energy exciton band and derivative features of higher energy transitions, which closely align with those observed in 400 nm-excited CdS NRs (Figure S14). At the same excitation fluence, negligible TA signals are observed for control samples of bare Cu_7_S_4_ NCs and bare CdS NRs (Figure S15). These results suggest the TA spectral features in Cu_7_S_4_/CdS NRs are caused by the interfacial transition from Cu_7_S_4_ VB to CdS CB instead of the plasmon-induced signal of the Cu_7_S_4_ domain or multiphoton absorption of CdS NRs. Similar TA spectra are also observed with 800 nm excitation (Figure S16), near the onset of interfacial charge transfer transition (∼1.5 eV, Figure S2b). Furthermore, the TA signal amplitude measured with 600 nm excitation increases linearly with the excitation fluence (Figure S17). These results indicate the photoexcitation at 600 and 800 nm directly transfers an electron from the Cu_7_S_4_ VB into the CB of CdS through DICTT (Figure 6b). We also fitted the XB kinetics at 473 nm for Cu_7_S_4_/CdS NRs under different pump fluences with a triexponential decay function according to eq 3. The fitting results are shown in Figure 6c. As shown in Table 1, τ_D1,XB_, τ_D2,XB_, and τ_D3,XB_ are determined to be 5.3 ± 1.1 ps, 102 ± 17.4 ps, and 5.6 ± 1.1 ns, respectively. These decay constants are very close to those from plasmon-induced indirect charge transfer (1400 nm pump), representing the same decay pathways for both directly transferred electrons and indirectly transferred electrons. This agreement can be seen in the comparison of the kinetics traces measured at 600 and 1400 nm, as shown in Figure 6d.

The comparison in Figure 6d also shows that the rise time of the exciton bleach measured at 600 nm excitation is instrument response time limited and is noticeably faster than that measured at 1400 nm excitation. This result is consistent with the direct charge transfer nature of the transition at 600 nm and suggests that the final state of the transition is at the CdS conduction band edge. The slower rise time of the XB kinetics measured by 1400 nm excitation of the plasmon band indicates negligible plasmon-induced hot electron transfer through the direct PICTT pathway, i.e., the coupling of the plasmon decay to the DICTT transition. This is consistent with a low hot electron QE at 1400 nm excitation. The lack of the PICTT pathway, despite the presence of the interfacial charge transfer transition, may be attributed to the energy mismatch of the DICTT band and plasmon band. Thus, reducing the energy mismatch between the plasmon and charge transfer transitions may present a fruitful direction for improving the plasmon-induced hot electron transfer efficiency.

## Conclusions

In summary, the present work investigates indirect and direct electron transfer in a Cu_7_S_4_/CdS heterojunction. Our results indicate that near-IR (1400 nm) excitation of the plasmon band in the Cu_7_S_4_ domain leads to indirect transfer of hot electrons to the CdS domain with an instrument response limited rise time of <150s fs and an estimated QE of ∼0.054%. The amplitude-weighted average lifetime of the transferred electrons in the Cu_7_S_4_/CdS heterostructure is 1.9 ± 0.5 ns, which is significantly longer than the ∼1.5 ps lifetime measured in Au/CdS nanoheterostructures. We attributed the much slower recombination time in the former to the presence of a band gap in Cu_7_S_4_, which slows the charge recombination process. Strong coupling between the Cu_7_S_4_ and CdS domains leads to the formation of direct interfacial charge transfer transition in the visible-near-IR region, and optical excitation of the DICTT at 600 and 800 nm can directly transfer an electron from the Cu_7_S_4_ VB to the CdS CB. Despite the presence of DICTT, the excitation of the plasmon band at 1400 nm does not lead to direct hot electron transfer through the PICTT pathway, likely due to the mismatch in the plasmon and DICTT energies. Achieving PICTT in plasmonic heterojunctions requires further material optimization, such as decreasing the DICTT transition energy and increasing the plasmon frequency to bring them closer resonance.

## Methods

CdS NRs were synthesized by following procedures reported in the literature with slight modifications.^38,51^ Both tips were then decorated with Cu_7_S_4_ NCs through the thermal decomposition of a Cu precursor, copper(II) dibutyldithiocarbamate. Details can be found in Note S1.

Instruments used for the structural and optical characterization of the NRs are also described in the Supporting Information. Femtosecond transient absorption (TA) measurements were conducted using a regeneratively amplified Ti:sapphire laser system (Coherent Legend, 800 nm, 150 fs, 3 mJ/pulse, and 1 kHz repetition rate). The TA signals were detected by Helios spectrometers and analyzed by using Surface Xplorer software (Ultrafast Systems LLC).

## Supporting Information Available

Details on sample synthesis, TEM images, additional absorption and TA spectra, TA kinetics comparison, global fitting results, band alignment estimation, hot electron transfer yield, lattice temperature calculations, and fitting parameters for transferred hot electrons and heating effects (PDF)

## References

[ref1] Zhang Y.; He S.; Guo W.; Hu Y.; Huang J.; Mulcahy J. R.; Wei W. D. Surface-Plasmon-Driven Hot Electron Photochemistry. Chem. Rev 2018, 118 (6), 2927–2954. 10.1021/acs.chemrev.7b00430. 29190069

[ref2] Narang P.; Sundararaman R.; Atwater H. A. Plasmonic hot carrier dynamics in solid-state and chemical systems for energy conversion. Nanophotonics 2016, 5 (1), 96–111. 10.1515/nanoph-2016-0007.

[ref3] Agrawal A.; Cho S. H.; Zandi O.; Ghosh S.; Johns R. W.; Milliron D. J. Localized Surface Plasmon Resonance in Semiconductor Nanocrystals. Chem. Rev 2018, 118 (6), 3121–3207. 10.1021/acs.chemrev.7b00613. 29400955

[ref4] Hartland G. V.; Besteiro L. V.; Johns P.; Govorov A. O. What’s so Hot about Electrons in Metal Nanoparticles?. ACS Energy Lett 2017, 2 (7), 1641–1653. 10.1021/acsenergylett.7b00333.

[ref5] Foerster B.; Joplin A.; Kaefer K.; Celiksoy S.; Link S.; Sonnichsen C. Chemical Interface Damping Depends on Electrons Reaching the Surface. ACS Nano 2017, 11 (3), 2886–2893. 10.1021/acsnano.6b08010. 28301133

[ref6] Yang W.; Liu Y.; Cullen D. A.; McBride J. R.; Lian T. Harvesting Sub-Bandgap IR Photons by Photothermionic Hot Electron Transfer in a Plasmonic p-n Junction. Nano Lett 2021, 21 (9), 4036–4043. 10.1021/acs.nanolett.1c00932. 33877837

[ref7] Lian Z.; Sakamoto M.; Vequizo J. J. M.; Ranasinghe C. S. K.; Yamakata A.; Nagai T.; Kimoto K.; Kobayashi Y.; Tamai N.; Teranishi T. Plasmonic p-n Junction for Infrared Light to Chemical Energy Conversion. J. Am. Chem. Soc 2019, 141 (6), 2446–2450. 10.1021/jacs.8b11544. 30563330

[ref8] Clavero C. Plasmon-induced hot-electron generation at nanoparticle/metal-oxide interfaces for photovoltaic and photocatalytic devices. Nat. Photonics 2014, 8 (2), 95–103. 10.1038/nphoton.2013.238.

[ref9] Brongersma M. L.; Halas N. J.; Nordlander P. Plasmon-induced hot carrier science and technology. Nat. Nanotechnol 2015, 10 (1), 25–34. 10.1038/nnano.2014.311. 25559968

[ref10] Liu G.; Lou Y.; Zhao Y.; Burda C. Directional Damping of Plasmons at Metal-Semiconductor Interfaces. Acc. Chem. Res 2022, 55 (13), 1845–1856. 10.1021/acs.accounts.2c00001. 35696292

[ref11] Ghorai N.; Ghosh H. N. Chemical Interface Damping in Nonstoichiometric Semiconductor Plasmonic Nanocrystals: An Effect of the Surrounding Environment. Langmuir 2022, 38 (18), 5339–5350. 10.1021/acs.langmuir.2c00446. 35491746

[ref12] Zhou D.; Li X.; Zhou Q.; Zhu H. Infrared driven hot electron generation and transfer from non-noble metal plasmonic nanocrystals. Nat. Commun 2020, 11 (1), 2944 10.1038/s41467-020-16833-1. 32522995 PMC7287091

[ref13] Chen Y.; Li Y.; Zhao Y.; Zhou H.; Zhu H. Highly efficient hot electron harvesting from graphene before electron-hole thermalization. Sci. Adv. 2019, 5 (11), eaax9958 10.1126/sciadv.aax9958. 31819905 PMC6884409

[ref14] Wen X.; Chen S.; Zhao J.; Du W.; Zhao W. Enhanced Plasmonic Hot-Carrier Transfer in Au/WS2 Heterojunctions under Nonequilibrium Condition. ACS Photonics 2022, 9 (5), 1522–1528. 10.1021/acsphotonics.1c01938.

[ref15] Yang W.; Liu Y.; McBride J. R.; Lian T. Ultrafast and Long-Lived Transient Heating of Surface Adsorbates on Plasmonic Semiconductor Nanocrystals. Nano Lett 2021, 21 (1), 453–461. 10.1021/acs.nanolett.0c03911. 33263400

[ref16] Wu K.; Rodriguez-Cordoba W. E.; Yang Y.; Lian T. Plasmon-induced hot electron transfer from the Au tip to CdS rod in CdS-Au nanoheterostructures. Nano Lett 2013, 13 (11), 5255–5263. 10.1021/nl402730m. 24093501

[ref17] Liu Y.; Chen Q.; Cullen D. A.; Xie Z.; Lian T. Efficient Hot Electron Transfer from Small Au Nanoparticles. Nano Lett 2020, 20 (6), 4322–4329. 10.1021/acs.nanolett.0c01050. 32374614

[ref18] Song J.; Long J.; Liu Y.; Xu Z.; Ge A.; Piercy B. D.; Cullen D. A.; Ivanov I. N.; McBride J. R.; Losego M. D.; Lian T. Highly Efficient Plasmon Induced Hot-Electron Transfer at Ag/TiO2 Interface. ACS Photonics 2021, 8 (5), 1497–1504. 10.1021/acsphotonics.1c00321.

[ref19] Wu K.; Chen J.; McBride J. R.; Lian T. Efficient hot-electron transfer by a plasmon-induced interfacial charge-transfer transition. Science 2015, 349 (6248), 632–635. 10.1126/science.aac5443. 26250682

[ref20] Wang C.; Jing Y.; Chen L.; Xiong W. Direct Interfacial Charge Transfer in All-Polymer Donor-Acceptor Heterojunctions. J. Phys. Chem. Lett 2022, 13 (37), 8733–8739. 10.1021/acs.jpclett.2c02130. 36095150 PMC9511559

[ref21] Tan S.; Argondizzo A.; Ren J.; Liu L.; Zhao J.; Petek H. Plasmonic coupling at a metal/semiconductor interface. Nat. Photonics 2017, 11 (12), 806–812. 10.1038/s41566-017-0049-4.

[ref22] Zhai L.; Gebre S. T.; Chen B.; Xu D.; Chen J.; Li Z.; Liu Y.; Yang H.; Ling C.; Ge Y. Epitaxial growth of highly symmetrical branched noble metal-semiconductor heterostructures with efficient plasmon-induced hot-electron transfer. Nat. Commun 2023, 14 (1), 2538 10.1038/s41467-023-38237-7. 37137913 PMC10156852

[ref23] Ostovar B.; Lee S. A.; Mehmood A.; Farrell K.; Searles E. K.; Bourgeois B.; Chiang W.-Y.; Misiura A.; Gross N.; Al-Zubeidi A.; Dionne J. A. The role of the plasmon in interfacial charge transfer. Sci. Adv. 2024, 10 (27), eadp3353 10.1126/sciadv.adp3353. 38968358 PMC11225779

[ref24] Le Formal F.; Pendlebury S. R.; Cornuz M.; Tilley S. D.; Gratzel M.; Durrant J. R. Back electron-hole recombination in hematite photoanodes for water splitting. J. Am. Chem. Soc 2014, 136 (6), 2564–2574. 10.1021/ja412058x. 24437340

[ref25] Zhai Y.; DuChene J. S.; Wang Y. C.; Qiu J.; Johnston-Peck A. C.; You B.; Guo W.; DiCiaccio B.; Qian K.; Zhao E. W. Polyvinylpyrrolidone-induced anisotropic growth of gold nanoprisms in plasmon-driven synthesis. Nat. Mater 2016, 15 (8), 889–895. 10.1038/nmat4683. 27376686

[ref26] Tang J.; Durrant J. R.; Klung D. R. Mechanism of Photocatalytic Water Splitting in TiO2. Reaction of Water with Photoholes, Importance of Charge Carrier Dynamics, and Evidence for Four-Hole Chemistry. J. Am. Chem. Soc 2008, 130 (42), 13885–13891. 10.1021/ja8034637. 18817387

[ref27] Luther J. M.; Jain P. K.; Ewers T.; Alivisatos A. P. Localized surface plasmon resonances arising from free carriers in doped quantum dots. Nat. Mater 2011, 10 (5), 361–366. 10.1038/nmat3004. 21478881

[ref28] Zhao Y.; Pan H.; Lou Y.; Qiu X.; Zhu J.; Burda C. Plasmonic Cu2– x S nanocrystals: optical and structural properties of copper-deficient copper (I) sulfides. J. Am. Chem. Soc 2009, 131 (12), 4253–4261. 10.1021/ja805655b. 19267472

[ref29] Xie Y.; Riedinger A.; Prato M.; Casu A.; Genovese A.; Guardia P.; Sottini S.; Sangregorio C.; Miszta K.; Ghosh S. Copper sulfide nanocrystals with tunable composition by reduction of covellite nanocrystals with Cu+ ions. J. Am. Chem. Soc 2013, 135 (46), 17630–17637. 10.1021/ja409754v. 24128337

[ref30] Liu Y.; Liu M.; Swihart M. T. Plasmonic Copper Sulfide-Based Materials: A Brief Introduction to Their Synthesis, Doping, Alloying, and Applications. J. Phys. Chem. C 2017, 121 (25), 13435–13447. 10.1021/acs.jpcc.7b00894.

[ref31] Dorfs D.; Hartling T.; Miszta K.; Bigall N. C.; Kim M. R.; Genovese A.; Falqui A.; Povia M.; Manna L. Reversible tunability of the near-infrared valence band plasmon resonance in Cu(2-x)Se nanocrystals. J. Am. Chem. Soc 2011, 133 (29), 11175–11180. 10.1021/ja2016284. 21728384

[ref32] Ghorai N.; Ghosh H. N. Ultrafast Plasmon Dynamics and Hole–Phonon Coupling in NIR Active Nonstoichiometric Semiconductor Plasmonic Cu2–xS Nanocrystals. J. Phys. Chem. C 2019, 123 (46), 28401–28410. 10.1021/acs.jpcc.9b10043.

[ref33] Wang J. J.; Xue D. J.; Guo Y. G.; Hu J. S.; Wan L. J. Bandgap engineering of monodispersed Cu(2-x)S(y)Se(1-y) nanocrystals through chalcogen ratio and crystal structure. J. Am. Chem. Soc 2011, 133 (46), 18558–18561. 10.1021/ja208043g. 22023550

[ref34] Liu Y.; Liu M.; Swihart M. T. Reversible Crystal Phase Interconversion between Covellite CuS and High Chalcocite Cu2S Nanocrystals. Chem. Mater 2017, 29 (11), 4783–4791. 10.1021/acs.chemmater.7b00579.

[ref35] Lian Z.; Sakamoto M.; Matsunaga H.; Vequizo J. J. M.; Yamakata A.; Haruta M.; Kurata H.; Ota W.; Sato T.; Teranishi T. Near infrared light induced plasmonic hot hole transfer at a nano-heterointerface. Nat. Commun 2018, 9 (1), 2314 10.1038/s41467-018-04630-w. 29899329 PMC5997981

[ref36] Lian Z.; Kobayashi Y.; Vequizo J. J. M.; Ranasinghe C. S. K.; Yamakata A.; Nagai T.; Kimoto K.; Kobayashi K.; Tanaka K.; Teranishi T.; et al. Harnessing infrared solar energy with plasmonic energy upconversion. Nature Sustainability 2022, 5, 1092 10.1038/s41893-022-00975-9.

[ref37] Lian Z.; Wu F.; Zhong Y.; Zi J.; Li Z.; Wang X.; Nakagawa T.; Li H.; Sakamoto M. Tuning plasmonic p–n junction for efficient infrared-light-responsive hydrogen evolution. Applied Catalysis B: environ 2022, 318, 121860 10.1016/j.apcatb.2022.121860.

[ref38] Liu Y.; Yang W.; Chen Q.; Cullen D. A.; Xie Z.; Lian T. Pt Particle Size Affects Both the Charge Separation and Water Reduction Efficiencies of CdS–Pt Nanorod Photocatalysts for Light Driven H2 Generation. J. Am. Chem. Soc 2022, 144, 2705 10.1021/jacs.1c11745. 35089025

[ref39] Alam R.; Labine M.; Karwacki C. J.; Kamat P. V. Modulation of Cu(2-x)S Nanocrystal Plasmon Resonance through Reversible Photoinduced Electron Transfer. ACS Nano 2016, 10 (2), 2880–2886. 10.1021/acsnano.5b08066. 26853633

[ref40] Wu K.; Li Q.; Jia Y.; McBride J. R.; Xie Z.-X.; Lian T. Efficient and Ultrafast Formation of Long-Lived Charge-Transfer Exciton State in Atomically Thin Cadmium Selenide/Cadmium Telluride Type-II Heteronanosheets. ACS Nano 2015, 9, 961–968. 10.1021/nn506796m. 25548944

[ref41] Li Q.; Lian T. Exciton Spatial Coherence and Optical Gain in Colloidal Two-Dimensional Cadmium Chalcogenide Nanoplatelets. Acc. Chem. Res 2019, 52 (9), 2684–2693. 10.1021/acs.accounts.9b00252. 31433164

[ref42] Li Q.; Wu K.; Zhu H.; Yang Y.; He S.; Lian T. Charge Transfer from Quantum-Confined 0D, 1D, and 2D Nanocrystals. Chem. Rev 2024, 124 (9), 5695–5763. 10.1021/acs.chemrev.3c00742. 38629390 PMC11082908

[ref43] Pandya R.; Chen R. Y. S.; Cheminal A.; Dufour M.; Richter J. M.; Thomas T. H.; Ahmed S.; Sadhanala A.; Booker E. P.; Divitini G. Exciton-Phonon Interactions Govern Charge-Transfer-State Dynamics in CdSe/CdTe Two-Dimensional Colloidal Heterostructures. J. Am. Chem. Soc 2018, 140 (43), 14097–14111. 10.1021/jacs.8b05842. 30293427

[ref44] Shabani F.; Martinez P. L. H.; Shermet N.; Korkut H.; Sarpkaya I.; Dehghanpour Baruj H.; Delikanli S.; Isik F.; Durmusoglu E. G.; Demir H. V. Gradient Type-II CdSe/CdSeTe/CdTe Core/Crown/Crown Heteronanoplatelets with Asymmetric Shape and Disproportional Excitonic Properties. Small 2023, 19 (11), e2205729 10.1002/smll.202205729. 36650974

[ref45] Hodak J. H.; Martini I.; Hartland G. V. Spectroscopy and Dynamics of Nanometer-Sized Noble Metal Particles. J. Phys. Chem. B 1998, 102, 6958–6967. 10.1021/jp9809787.

[ref46] Blemker M. A.; Gibbs S. L.; Raulerson E. K.; Milliron D. J.; Roberts S. T. Modulation of the Visible Absorption and Reflection Profiles of ITO Nanocrystal Thin Films by Plasmon Excitation. ACS Photonics 2020, 7 (5), 1188–1196. 10.1021/acsphotonics.9b01825.

[ref47] Lou Y.; Samia A. C. S.; Cowen J.; Banger K.; Chen X.; Lee H.; Burda C. Evaluation of the photoinduced electron relaxation dynamics of Cu1.8S quantum dots. Phys. Chem. Chem. Phys 2003, 5 (6), 1091–1095. 10.1039/b211104g.

[ref48] Cooper J. K.; Reyes-Lillo S. E.; Hess L. H.; Jiang C.-M.; Neaton J. B.; Sharp I. D. Physical Origins of the Transient Absorption Spectra and Dynamics in Thin-Film Semiconductors: The Case of BiVO4. J. Phys. Chem. C 2018, 122 (36), 20642–20652. 10.1021/acs.jpcc.8b06645.

[ref49] Wu K.; Zhu H.; Liu Z.; Rodriguez-Cordoba W.; Lian T. Ultrafast charge separation and long-lived charge separated state in photocatalytic CdS-Pt nanorod heterostructures. J. Am. Chem. Soc 2012, 134 (25), 10337–10340. 10.1021/ja303306u. 22655858

[ref50] Jen-La Plante I.; Teitelboim A.; Pinkas I.; Oron D.; Mokari T. Exciton Quenching Due to Copper Diffusion Limits the Photocatalytic Activity of CdS/Cu2S Nanorod Heterostructures. J. Phys. Chem. Lett 2014, 5 (3), 590–596. 10.1021/jz500041g. 26276614

[ref51] Liu Y.; Yang W.; Chen Q.; Xie Z.; Lian T. Nanorod length-dependent photodriven H2 production in 1D CdS–Pt heterostructures. J. Chem. Phys 2023, 159 (10), 104706 10.1063/5.0157927. 37698197

