# Supplementary material for: Direct and Indirect Interfacial Electron Transfer at a Plasmonic p-Cu7S4/CdS Heterojunction
Source: ACS Nano. 2025 Jan 2;19(1):1547–56. doi: 10.1021/acsnano.4c14556 (PMC11752524; doi:10.1021/acsnano.4c14556)
Supplement: Supplementary file 1 — nn4c14556_si_001.pdf [file nn4c14556_si_001.pdf]

**Supporting information**

# Direct and Indirect Interfacial Electron Transfer at a Plasmonic p-$Cu_7S_4$/CdS Heterojunction

Zhicheng Yang, Nandan Ghorai, Shengxiang Wu, Sheng He, Tianquan Lian

**Note S1.** Sample Preparation and Characterization
**Note S2.** Estimation of the Band Alignment in The Heterostructure
**Note S3.** Estimation of the Hot Electron Transfer Yield (1400 nm pump)
**Note S4.** Estimation of the Temperature for $Cu_7S_4$/CdS NRs
**Note S5.** Fitting Parameters for Transferred Hot Electrons and the Heating Effect

**Figure S1.** TEM and Morphological Analysis
**Figure S2.** Steady State Absorption
**Figure S3.** TA Spectra for Bare CdS NRs
**Figure S4.** Spectral and Kinetic Comparison of Bare $Cu_7S_4$ and $Cu_7S_4$/CdS Heterojunction
**Figure S5.** UV-vis Absorption Spectra at Varying Temperatures
**Figure S6.** Linear Dependence of Heat-induced Signals
**Figure S7.** Comparison for Heat-induced Spectra
**Figure S8.** TA Spectra for Bare $Cu_7S_4$ with 400 nm Excitation
**Figure S9.** Shape Comparison for Bare CdS and $Cu_7S_4$/CdS with 400 nm Excitation
**Figure S10.** Shape Comparison for Heat-induced Spectra
**Figure S11.** Fitting for The Extracted Spectra (1400 nm excitation)
**Figure S12.** Two-component Fitting Results
**Figure S13.** Kinetic Comparison for Band Edge Electrons
**Figure S14.** Shape Comparison of 400 nm-excited CdS NRs and 600 nm-excited $Cu_7S_4$/CdS NRs.
**Figure S15.** Spectra for bare $Cu_7S_4$ NCs and CdS NRs (600 nm excitation)
**Figure S16.** Spectra for $Cu_7S_4$, CdS, and $Cu_7S_4$/CdS (800 nm excitation)
**Figure S17.** Fluence-dependence for XB in $Cu_7S_4$/CdS (600 nm excitation)

# Note S1. Sample Preparation and Characterization

## Reagents

Cadmium oxide (CdO, 99.998%), 1-octadecene (ODE, 90%), oleic acid (OA, 90%), sulfur power (S, 99.999%), trioctylphosphine oxide (TOPO 99%), trioctylphosphine (TOP, 97%), oleylamine (OLA, 98%), oleylamine (OLA, 70%), and 1-dodecanethiol (DDT, 98%) were purchased from Sigma Aldrich. 1,2-hexadecanediol, N-hexylphosphonic acid (HPA, 95%), octadecylphosphonic acid (ODPA, 97%), and Copper(II) Dibutyldithiocarbamate ($Cu(S_2CNBut_2)_2$) were purchased from TCI chemicals. All chemicals were used without further purification.

## Synthesis of CdS nanorods (NRs)

CdS NRs were synthesized according to a reported seeded-growth method with modifications[1]. To prepare the CdS quantum seeds, 60 mg CdO, 6 mL 1-octadecene (ODE) and 0.45 mL oleic acid (OA) loaded in a 50 mL three-neck flask was degassed under 150 $^{o}$C and then purged with Ar under 260 $^{o}$C. After the full dissolution of CdO at 260 $^{o}$C under stirring, sulfur dissolved in ODE (0.8mL, 8 mg/mL) was injected. The reaction was allowed to proceed for 40 seconds, followed by putting the flask into a room temperature water bath to quench the reaction. The products were washed three times by precipitation/redispersion with ethanol/toluene. Products are finally dispersed in toluene for later use. The first exciton peak of CdS quantum seeds is at around 375 nm.

In a typical synthesis of CdS NRs, 0.12 g cadmium oxide (CdO), 4.5 g TOPO, 0.45 g ODPA, and 0.12 g HPA were added into a 25 mL three-necked flask, which was then degassed under vacuum for 1 h at 150 $^{o}$C. After that, the flask was heated to 350 $^{o}$C under argon. Gradually, the mixture in the flask turned into clear solution, indicating the full dissolution of CdO. At this point, 1.8 mL trioctylphosphine (TOP) was injected to the solution. After the temperature of the reaction solution recovered to and stabilized at 350 $^{o}$C, sulfur stock solution (0.12g) with CdS quantum seeds (~80 nmol) in 1.8 mL TOP was injected. The temperature of the solution recovered to 350 $^{o}$C within ~4 min. The reaction was allowed to proceed for another ~4 min (total 8 min) and then stopped by removing the heating mantle and cooled down with compressed air. Crude solution was distributed equally into two centrifuge tubes and precipitated by the addition of toluene (~4 mL) and ethanol (~2 mL) in each tube. The yellow supernatant containing CdS nanoparticles with bad shapes was discarded and yellow precipitate was dispersed in toluene. The dissolution and precipitation processes were repeated with toluene and ethanol twice. Finally, the CdS NRs were dispersed in toluene.

## Synthesis of $Cu_7S_4$ nanocrystals (NCs)

The synthesis of $Cu_7S_4$ nanocrystal (NC) is similar as previous report[2]. In brief, 4.0 mL of OLA (98%), 6.0 mL of ODE, and 0.08 mL of DDT were mixed in a reaction flask and gradually heated to 205 °C under a gentle argon flow with magnetic stirring. Then 16 mg of $Cu(S_2CNBut_2)_2$ was sonicated in 1.0 mL of OLA (70%) for 5 minutes and the mixed solution was injected into the flask. After keeping the temperature at 190 °C for 10 min, the result solution was cooled to room

temperature. The crude solution was centrifuged with adding extra ethanol and hexane. The final product was dispersed in chloroform.

**Synthesis of $Cu_7S_4$/CdS heterostructure**

The synthesis of $Cu_7S_4$ /CdS NC is similar as that of $Cu_7S_4$ NC. The only difference is certain amount of CdS NRs was added to the injection solution. In detail, CdS NR solution in toluene (OD=10 @ first exciton peak with 1 cm path length, volume = 1 mL) was evaporated and re-dispersed in 1 mL OLA (70%) with $Cu(S_2CNBut_2)_2$, then the mixture was sonicated for 3 minutes.

**Transient absorption Setup**

The transient absorption measurements were conducted in a Helios spectrometer (Ultrafast Systems LLC). The 800 nm fundamental light was generated by a Ti: Sapphire laser (Coherent Legend, 150 fs, 3 mJ/pulse, and 1 kHz repetition rate) and split into two parts. One part of 800 nm beam was attenuated and focused on a sapphire window for the generation of the white light continuum. Probe beams were focused into a fiber optics-coupled multichannel spectrometer with complementary metal-oxide-semiconductor (CMOS) sensors and detected at a frequency of 1 kHz. Another part of the 800 nm beam is focused into a frequency doubling BBO crystal to generate the 400 nm pump excitation. For the case of 1400 nm excitation, the 800 nm was focused into an optical parametric amplifier (Light conversion, TOPAS) and we utilized a signal beam for the generation of 1400 nm pump after the pre-amplification stage. In both cases, the pump beam was chopped at 500 Hz. The delay time between the pump and probe pulse was controlled by a motorized delay stage. The change in the transmitted probe pulses for the pumped and unpumped samples was calculated and converted to the absorption difference. The samples were dispersed in cuvette and stirred vigorously during the measurement.

It should be noted that samples measured here were dispersed in a mixed solution of chloroform and DDT (1:1 in volume) and kept in 1 mm length quartz cuvettes. Herein, DDT is introduced as the hole scavenger trapping generated holes.

**Absorption spectroscopy and the temperature-dependent absorption**

The UV-vis absorption spectra were recorded on Agilent 8453 spectrometer. The UV-vis-NIR absorption spectra were recorded on Shimadzu 3600 spectrometer.

The temperature dependent UV-vis absorption spectra were measured on Horiba Dual-FL spectrometer, equipped with a Peltier temperature control unit (Newport, LDT-5525B). The samples were dispersed and measured in chloroform in a 1 cm cuvette. The temperature of the samples was ramped from 15–40 $^oC$ in a 5 $^oC$ interval. After reaching each defined temperature, the samples were allowed to equilibrate for 3 mins to stabilize its temperature.

**Transmission electron microscopy**

Transmission electron microscopy (TEM) images were acquired on a Hitachi HT7700 with an acceleration voltage of 80 kV to obtain morphology information.

## Note S2. Estimation of the Band Alignment in the Heterostructure

We estimate the band-edge electron and hole energy ($E_{1\sigma_e}$and $E_{1\sigma_h}$) of the CdS NR using a previously reported procedure.[3]

$$E_{1\sigma_e} = E_{CB} + \frac{m_h}{m_h+m_e}[E_g(NR) + V_{e-h} - E_g(bulk)] \qquad \text{(S1)}$$
$$E_{1\sigma_h} = E_{VB} - \frac{m_e}{m_h+m_e}[E_g(NR) + V_{e-h} - E_g(bulk)] \qquad \text{(S2)}$$

Here $E_{CB}$ and $E_{VB}$ represent the conduction and valence band edge energy of the bulk CdS, respectively. $E_g(bulk)$ represents the bulk bandgap of CdS. $m_e$ and $m_h$ represent the bulk effective mass of CdS electron and hole, respectively. $E_g(NR)$ represents the lowest exciton energy of CdS NRs. $V_{e-h}$ stands for the electron-hole columbic interaction energy.

Using the parameters listed in Table S1, the conduction and valence band of the CdS NRs can be estimated to be –3.52 and -6.42 V (vs vacuum, same for below), respectively.

**Table S1:** Parameters to estimate the band-edge energy of CdS NR

| Parameters | Values |
|---|---|
| $\boldsymbol{E_{CB}}$ | -3.84 V[4] |
| $\boldsymbol{E_{VB}}$ | -6.34 V[4] |
| $\boldsymbol{V_{e-h}}$ | 0.19 V[5] |
| $\boldsymbol{E_g(bulk)}$ | 2.5 V[4] |
| $\boldsymbol{E_g(NR)}$ | 2.7 V |
| $\boldsymbol{m_e}$ | 0.19 $m_0$ [4] |
| $\boldsymbol{m_h}$ | 0.8 $m_0$ [4] |

For $Cu_7S_4$, Cui *et al.* determined the position of the valence band and Fermi level to be ~ -5.32 eV and ~ -5.19 eV, respectively.[6] We further estimate the optical bandgap from the band edge absorption of the UV-vis absorption spectra, which is ~2.14 eV. With this data, we estimate the position of the conduction band edge to be -3.18 eV. Thus, the conduction band offset between CdS and $Cu_7S_4$ can be expected to be 0.34 eV, forming a type II or quasi-type II heterostructure. This band alignment agrees with previous studies of $Cu_{2-x}S$/CdS heterostructure.[7, 8] It should be noted that a small variation of the estimated values does not affect the band alignments and discussion in this study.

## Note S3. Estimation of the Hot Electron Transfer Yield (1400 nm pump)

In the TA measurement, under the low averaged electron number conditions, the XB amplitude of CdS NRs scales linearly with the number of electrons in its conduction band regardless the electrons are directly generated by 400 nm photoexcitation or transferred from the $Cu_7S_4$ domain after 1400 nm excitation.

Therefore, to characterize the number of transferred electrons from $Cu_7S_4$ to CdS under 1400 nm photoexcitation, we first characterize the relationship between the absorbed photons density of CdS ($N_{400}$) and the resulting XB amplitude ($\Delta A_{XB}$) using 400 nm photoexcitation in pure CdS NRs. Then, we use the extracted XB amplitude at 3 ps under 1400 nm excitation to characterize the number density of transferred electrons in the CdS domain ($N_{400,\ HET}$) by equation (S3):

$$N_{400,HET} = \frac{\Delta A_{XB,HET}}{\Delta A_{XB}} N_{400} \qquad \text{(S3)}$$

Hence, one can estimate the hot electron transfer yield by equation (S4),

$$\eta = \frac{N_{400,HET}}{N_{1400}} * 100\% \qquad \text{(S4)}$$

$N_{400}$ $and$ $N_{1400}$ are the absorbed photon densities of the CdS nanorods at 400nm and the $Cu_7S_4$/CdS heterostructures at 1400 nm, respectively, which can be described by:

$$N_{400} = \frac{pulse\ energy\ (400nm)/hv}{pump\ beam\ size\ (400\ nm) * \boldsymbol{l_{400nm}}} * (1 - 10^{-OD(\text{CdS},400\ nm)}) \qquad \text{(S5)}$$

$$N_{1400} = \frac{pulse\ energy\ (1400\ nm)/hv}{pump\ beam\ size\ (1400\ nm) * \boldsymbol{l_{1400nm}}} * (1 - 10^{-OD(\text{Cu7S4/CdS},1400\ nm)}) \qquad \text{(S6)}$$

Here pump pathlength at both 400nm and 1400nm are assumed to be the sample cell pathlength (1mm). The detailed parameters are shown in Table S2. Using these parameters, the hot electron transfer quantum efficiency can be calculated to be 0.054% under our experimental conditions.

**Table S2:** parameters for TA experiments.

| Excitation wavelength (nm) | Pump pulse power | hν (eV) | Pump Beam ($\mu m^2$) | Optical Density (OD) (L=1mm, data not shown) | $\Delta A$ (mOD) |
|---|---|---|---|---|---|
| 400 (CdS) | 1.4 μW<br>$2.8*10^{-9}$J | 3.1 | 19327.1±4276.5 | 0.34 | 3.81 |
| 1400 ($Cu_7S_4$/CdS) | 1 mW<br>$2*10^{-6}$J | 0.89 | 37899.8±3811.00.03 | 0.26 | 2.18 |

## Note S4. Estimation of the Temperature for $Cu_7S_4$/CdS NRs

For the steady state heating measurement, the absorbance of the sample is given by:

$$\boldsymbol{A_{steady}(\lambda, T) = \varepsilon(\lambda, T) \cdot c \cdot l} \qquad \text{(S7)}$$

In Eq. (S7), $\varepsilon(\lambda, T)$ is the wavelength and temperature dependent extinction coefficient, c the sample concentration, and l sample pathlength. Temperature dependence absorbance change is given by:

$$\boldsymbol{\Delta A(\lambda, \Delta T) = A(\lambda, T_2) - A(\lambda, T_1) = [\varepsilon(\lambda, T_2) - \varepsilon(\lambda, T_1)] \cdot c \cdot l} \qquad \text{(S8)}$$

Because we observed linear dependence of absorbance change on temperature change, it can be assumed that $\boldsymbol{\varepsilon(\lambda, T_2) - \varepsilon(\lambda, T_2) = \alpha(\lambda) \cdot \Delta T}$, where $\boldsymbol{\alpha(\lambda)}$ is a proportionality constant. From the steady state temperature dependent spectra, we can determine the $\boldsymbol{\alpha(\lambda)}$ value according to Eq. (S9).

$$\boldsymbol{\alpha(\lambda) = \frac{\Delta A(\lambda, \Delta T)}{\Delta T \cdot c \cdot l}} \qquad \text{(S9)}$$

Herein, the concentration of NRs can be estimated to be $8.4 * 10^{12}\ cm^{-3}$ by Eq. (S10).

$$\boldsymbol{c_{NRs}} = \frac{A_{350nm}}{\varepsilon_{350nm} * l} \qquad \text{(S10)}$$

where $\varepsilon_{350nm}$ is determined to be $2.2 * 10^7\ M^{-1}cm^{-1}$ from Eq. (S11) with $V_{CdS}$ = 790 $nm^3$ determined from Fig. S1.

$$\varepsilon_{CdS,350\ nm} = 2.8 * 10^{25} M^{-1}cm^{-4} * V. \qquad \text{(S11)}$$

For $Cu_7S_4$/CdS NRs, the $\alpha(482\ nm)$ is calculated to be $5.7 * 10^{-14}\ mOD * K^{-1} * cm^2$.

In the transient absorption measurement, the concentration of absorbed 1400 nm photon per pulse is estimated to be $1.7 * 10^{14}\ mm^{-3}$ from Eq. (S12).

$$\boldsymbol{c_{photon}} = \frac{pulse\ energy\ (\upsilon_{pump})\left(1-10^{-OD(\upsilon_{pump})}\right)}{beam\ size\ (\upsilon_{pump}) h\upsilon_{pump} l_{pump}} \qquad \text{(S12)}$$

On the other hand, the NRs' concentration is determined to $2.0 * 10^{11}\ mm^{-3}$ for TA experiment. Due to $\boldsymbol{c_{photon}} \gg \boldsymbol{c_{NRs}}$, the concentration of excited NRs $\boldsymbol{c^*}$ can be represented by $\boldsymbol{c_{NRs}}$**.** The measured absorbance change of the excited sample caused by heating is given by:

$$\boldsymbol{\Delta A(t, \lambda) = A(T_{ex}, \lambda) - A(T_0, \lambda) = \alpha(\lambda) \cdot \Delta T(t) \cdot c^* \cdot l_{probe} \cdot \frac{l_{probe}}{l_{pump}}}$$
$$\boldsymbol{= \alpha(\lambda) \cdot \Delta T(t) \cdot c_{NRs} \cdot l_{probe} \cdot cos\theta} \qquad \text{(S13)}$$

In Eq. (13), $\boldsymbol{l_{pump}}$ is length of the excited sample region and $\boldsymbol{l_{probe}}$ is the length of the excited sample region probed in the measurement and the ratio of these two quantities depends on the angel between the pump and probe beam, $\frac{l_{probe}}{l_{pump}} = cos\theta$. In our experiment, the pump/probe is ~ $6.6^{o}$. Using Eq. (S13), we can estimate the temperature increase of the sample from the measured absorbance change in the heating induced TA spectral component.

Using the parameters in Table S3, we estimate that the temperature increases by 0.38 K, which is similar to the rise in lattice temperature simulated in bare $Cu_{2-x}Se$ and Au under similar excitation fluence. [9]

**Table S3:** parameters to estimate the lattice temperature.

| | $\Delta A$ | $\Delta T$ | $c^*$ | $l$ |
|---|---|---|---|---|
| Temperature-dependent experiment | 26.7 mOD (Fig. 3d, purple curve at 482 nm) | 25.0 K | $1.9 * 10^{13}\ cm^{-3}$ | 10 mm |
| TA experiment | 0.42 mOD (Fig. S11d, blue curve at 482 nm) | 0.38 K | $2.0 * 10^{14}\ cm^{-3}$ | 1 mm |

**Note S5.** Fitting Parameters for Transferred Hot Electrons and the Heating Effect

The kinetics for transferred hot electrons and the heating effect (Fig. 5c) are fitted by Eq. 2 and Eq. 4 in the main text, respectively.

**Table S4:** fitting parameters for hot electron cooling and heat transfer in the CdS domain.

| | Rising Constant (ps) | Decay Constant (ps) |
|---|---|---|
| Hot electron | IRF limited | 0.19 ± 0.07 |
| Heat transfer | 22.6 ± 2.6 | 972.7 ± 87.2 |

**Figure S1. TEM and Morphological Analysis**

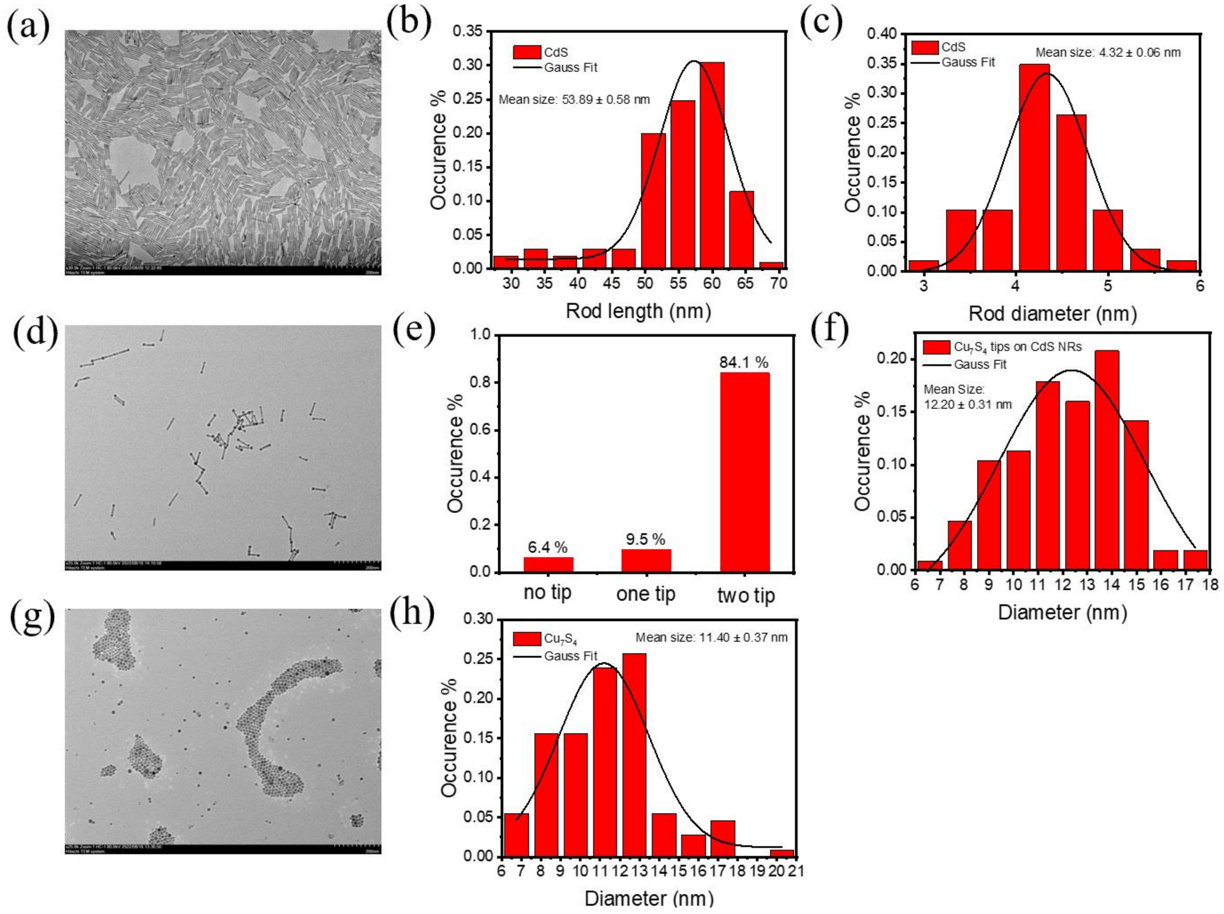


**Fig. S1** (a) TEM image of CdS NRs. (b) and (c) are length and diagram distributions of CdS NRs, respectively. (d) TEM image of $Cu_7S_4$/CdS NRs. (e) Percentage of $Cu_7S_4$/CdS NRs with none, one, or $Cu_7S_4$ two tips. (f) size distribution of $Cu_7S_4$ domain in $Cu_7S_4$/CdS NRs. (g) TEM image of $Cu_7S_4$ NCs. (h) size distribution of free $Cu_7S_4$ NCs.

**Figure S2. Steady State Absorption**

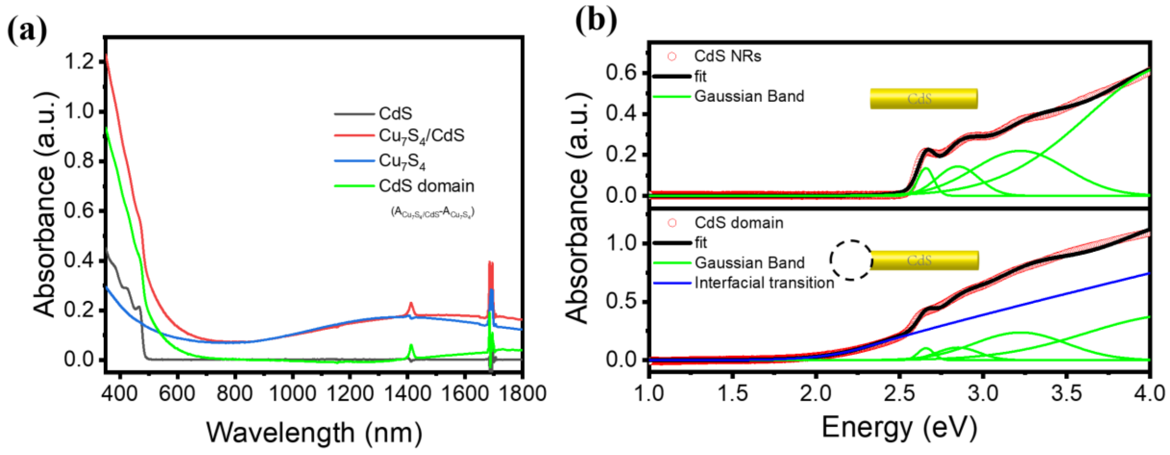


**Fig. S2** (a) UV-vis-NIR absorption spectra for a new series of samples. The plasmon bands for the bare $Cu_7S_4$ and the $Cu_7S_4$ domain are similar. (b) Comparison for the bare CdS NRs and CdS domain. The spectra of CdS domain are obtained by subtracting $Cu_7S_4$ spectra from the $Cu_7S_4$/ CdS spectra.

**Figure S3. TA Spectra for Bare CdS NRs**

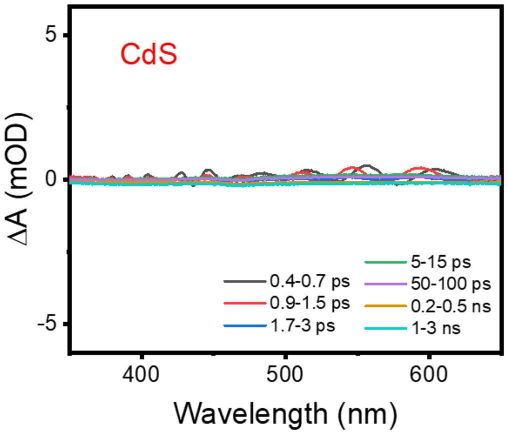


**Fig. S3** TAs of CdS NRs after 1400 nm excitation. $Cu_7S_4$ NC (Fig. 2a) and $Cu_7S_4$/CdS NRs (Fig. 2b) were performed under the same condition (1.33 mJ/cm$^2$ ).

**Figure S4. Spectral and Kinetic Comparison of Bare $Cu_7S_4$ and $Cu_7S_4$/CdS Heterojunction.**

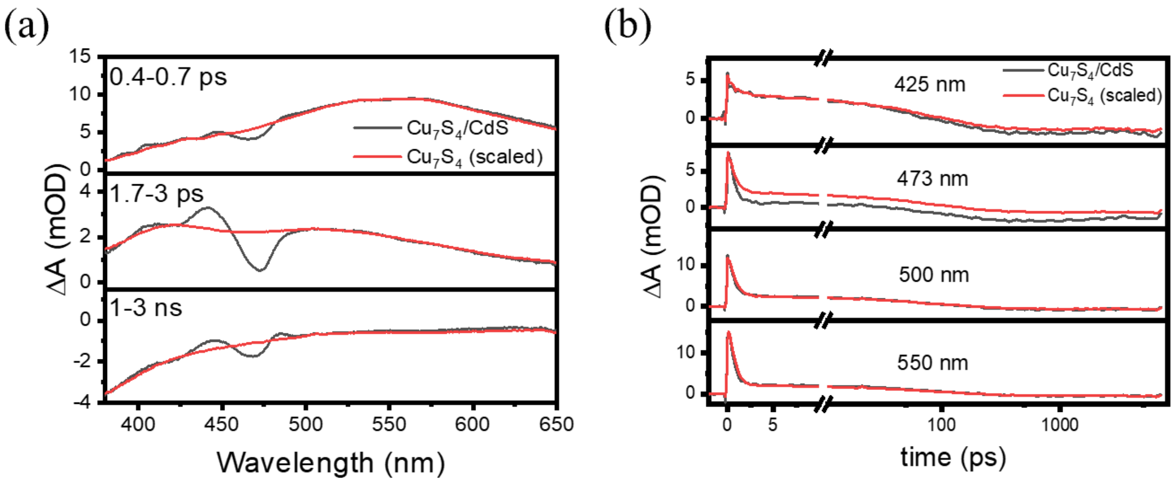


**Figure S4.** (a) Comparison of the spectra for $Cu_7S_4$ NCs (red line) and $Cu_7S_4$/CdS NRs (black line) at different delay times. The spectra of $Cu_7S_4$ NCs are scaled for ease of comparison. (b) Comparison of the kinetics for $Cu_7S_4$ NCs (red line) and $Cu_7S_4$/CdS NRs (black line) at different wavelengths. The kinetic trace for $Cu_7S_4$ NCs is scaled for ease of comparison.

**Figure S5. UV-vis Absorption Spectra at Varying Temperatures.**

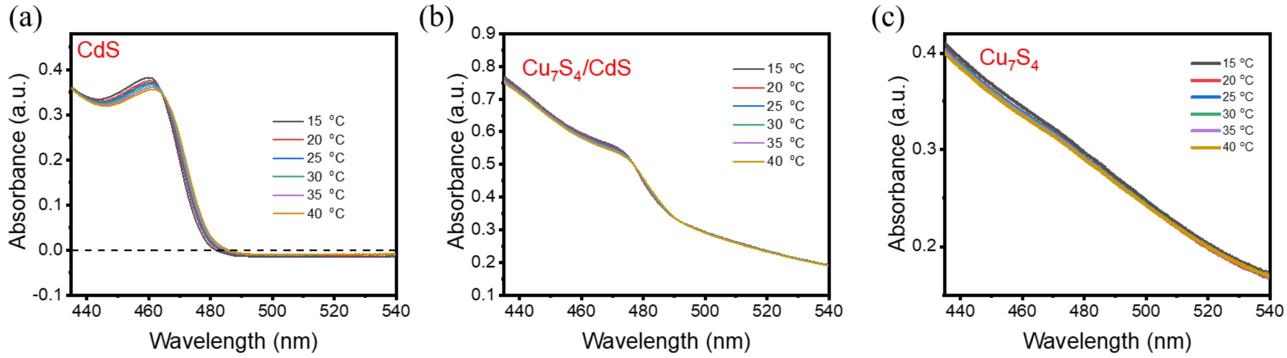


**Fig. S5** UV-vis absorption spectra of (a) CdS NRs, (b) $Cu_7S_4$ NC, and (c) $Cu_7S_4$/CdS NRs when the temperature was ramped from 15 to 40 $^oC$ at 5 $^oC$ intervals.

**Figure S6. Linear Dependence of Heat-induced Signals.**

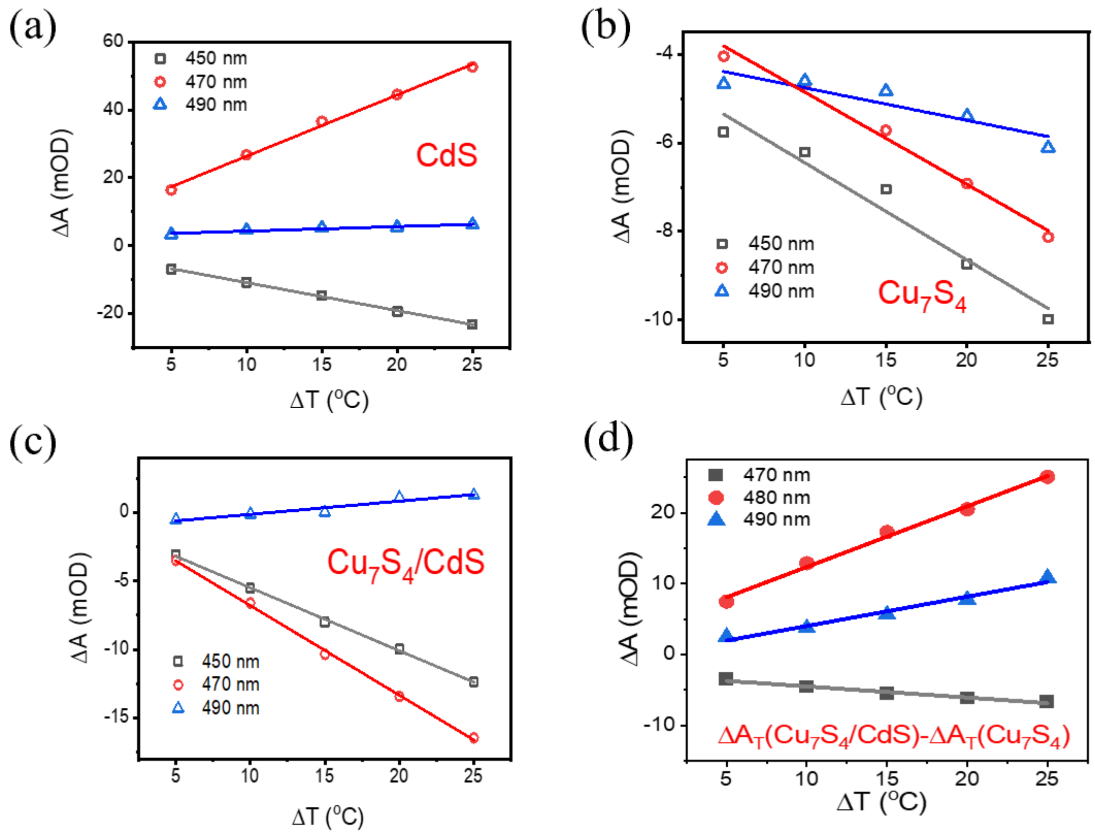


**Fig. S6** The heat-induced amplitude difference of CdS (a), $Cu_7S_4$ (b), $Cu_7S_4$/CdS (c), and the CdS domain (d) are linearly dependent on temperature differences.

**Figure S7. Comparison for Heat-induced Spectra.**

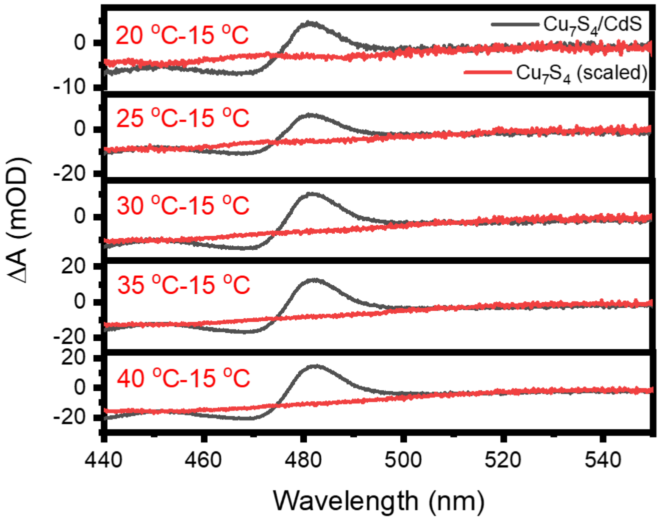


**Fig. S7** Comparison of heat-induced difference absorption spectra of $Cu_7S_4$ NCs and $Cu_7S_4$/CdS NRs at various temperatures.

**Figure S8. TA Spectra for Bare $Cu_7S_4$ with 400 nm Excitation.**

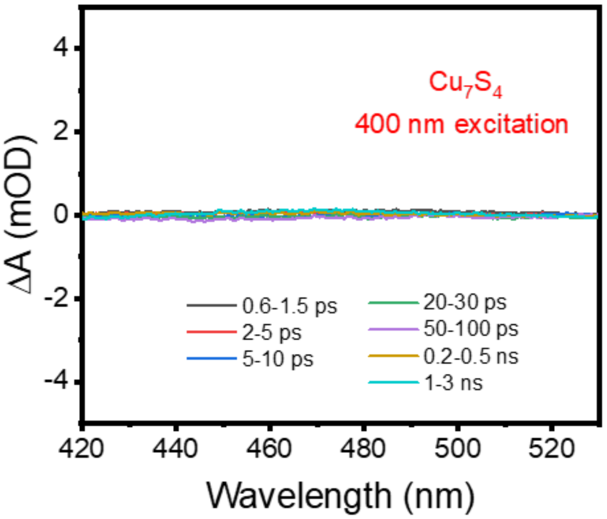


**Fig. S8** TA Spectra for Bare $Cu_7S_4$ with 400 nm Excitation. No signal is observed in our experiment.

**Figure S9. Shape Comparison for Bare CdS and $Cu_7S_4$/CdS with 400 nm Excitation.**

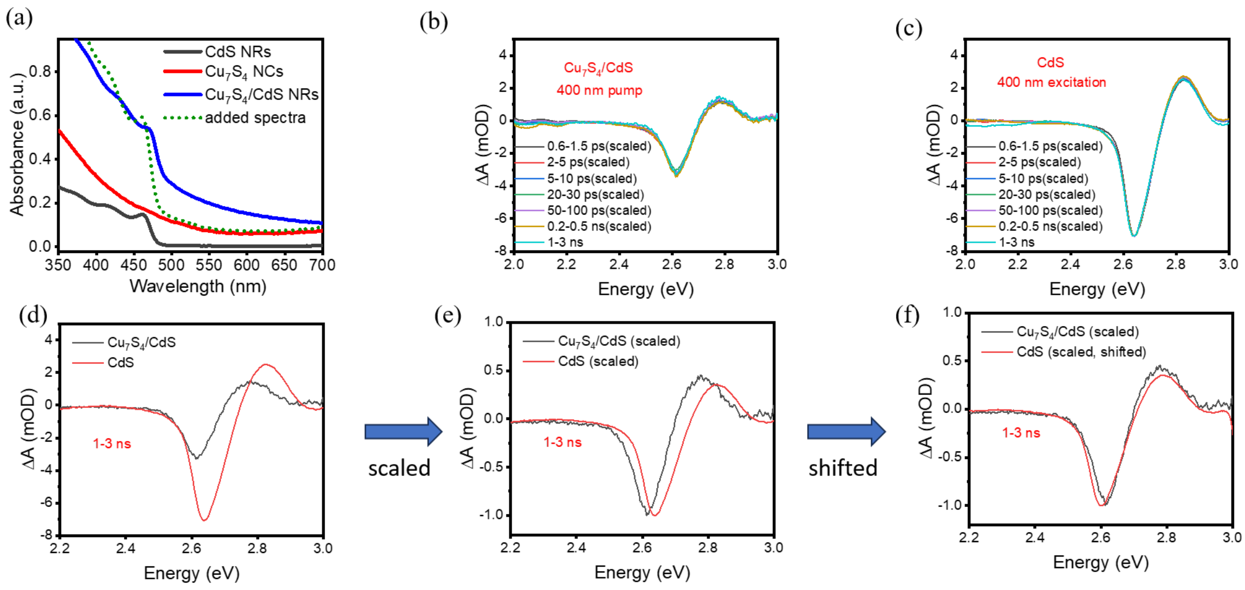


**Fig. S9** (a) A zoomed-in view of Figure 1d. The CdS exciton peak in $Cu_7S_4$/CdS (blue solid curve) undergoes a red shift compared to the exciton peak in the added spectrum (green dotted curve), which contains the CdS NR contribution before the $Cu_7S_4$ growth. Scaled TA Spectra for $Cu_7S_4$/CdS (b) and bare CdS (c) with 400 nm Excitation. Spectra at different delay times can overlap very well after scaling. After scaling and shifting, the spectra for $Cu_7S_4$/CdS and bare CdS

collected at 1-3 ns are in good agreement (d-f). The amplitude scaling is needed because these samples have different optical densities at the excitation wavelength (400nm). Furthermore, the diameter of the CdS nanorod in CdS only and in $Cu_7S_4$/CdS samples are slightly different, which shifts the CdS exciton position, as shown in Figure S9a. For this reason, the CdS TA spectra were shifted for better comparison.

**Figure S10. Shape Comparison for Heat-induced Spectra.**

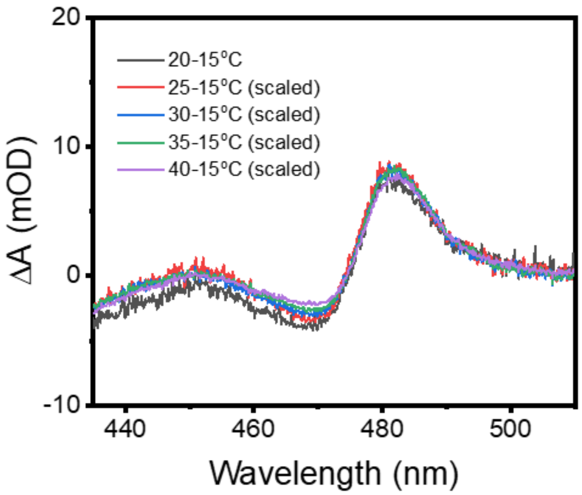


**Fig. S10** Comparison of extracted heat-induced difference absorption spectra of CdS domain in $Cu_7S_4$/CdS NRs. Spectra are from Fig. 3d and scaled for ease of comparison.

**Figure S11. Fitting for the extracted spectra (1400 nm excitation).**

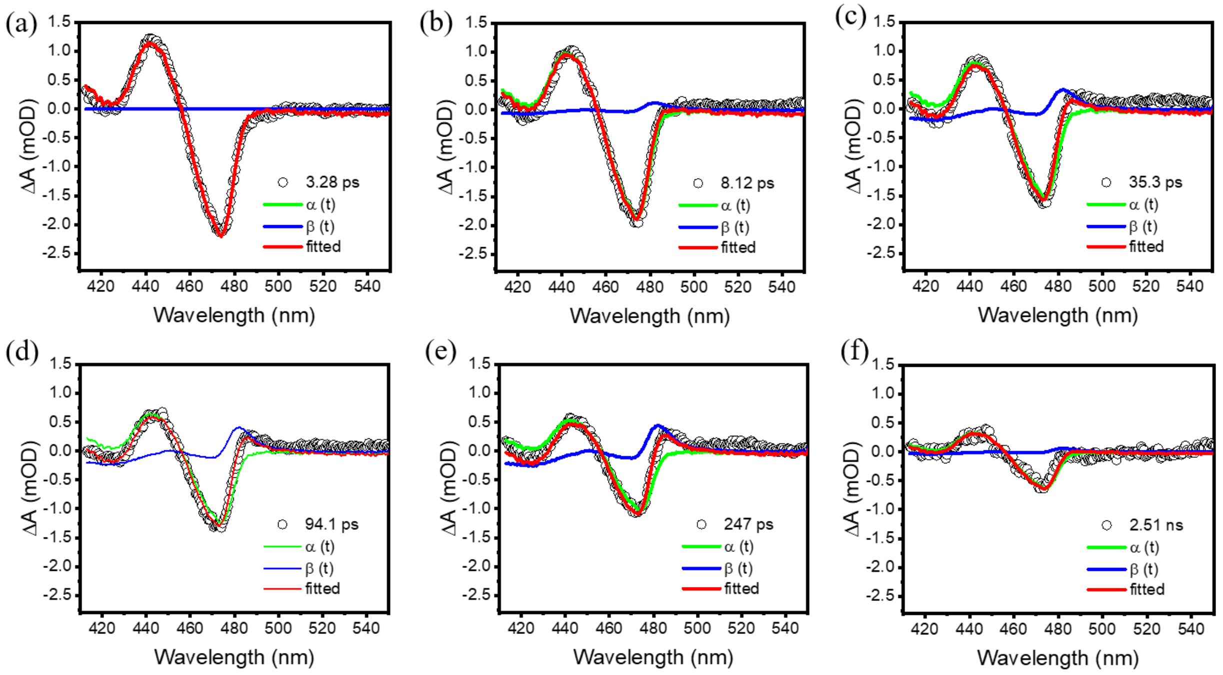


**Fig. S11.** Global fitting examples for single delay times of the extracted spectra of the CdS domain in $Cu_7S_4$/CdS NRs obtained by subtracting the scaled $Cu_7S_4$ spectra in panel a from the $Cu_7S_4$/CdS spectra in panel b of Figure 2.

**Figure S12. Two-component fitting results of the extracted spectra of the CdS domain in $Cu_7S_4$/CdS NRs.**

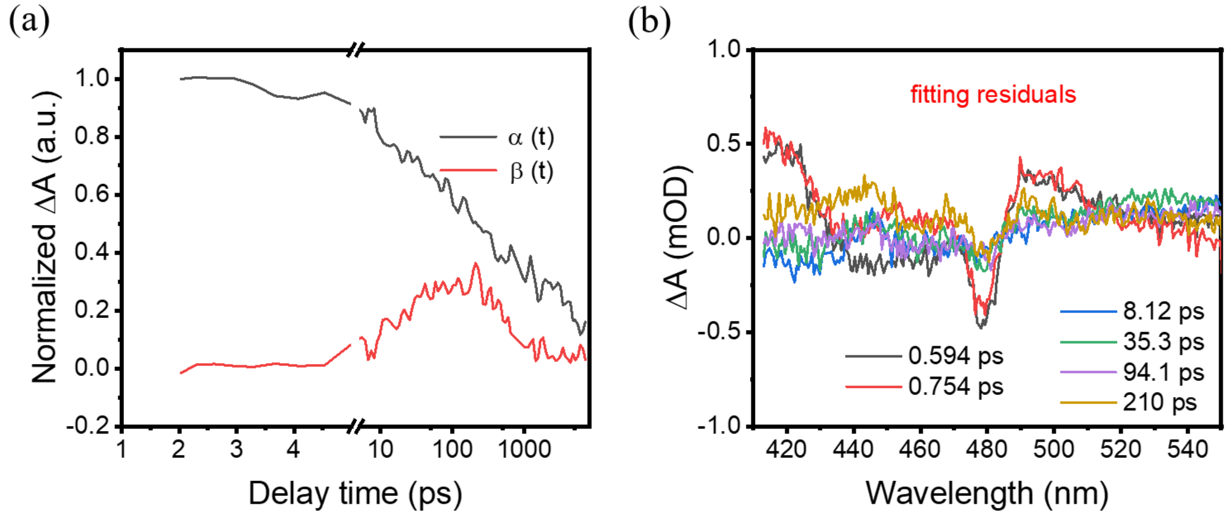


**Fig. S12**. (a) Time-dependent amplitude change for band edge electron ($\alpha$) and heat effect ($\beta$). Transferred hot electron contributes to $\beta$ at early times; hence, the fitting is valid after 2 ps. (b) The fitting residuals.

**Figure S13. Kinetic Comparison for Band Edge Electrons.**

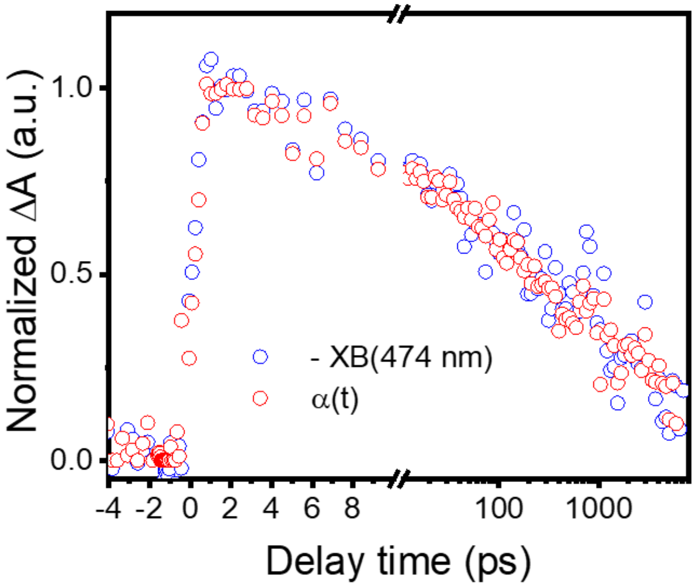


**Fig. S13**. Blue open circles: kinetic obtained 474 nm. Red open circles: time dependent amplitude change for band edge electron (from two-component fitting).

**Figure S14.** Shape Comparison of 400 nm-excited CdS NRs and 600 nm-excited $Cu_7S_4$/CdS NRs.

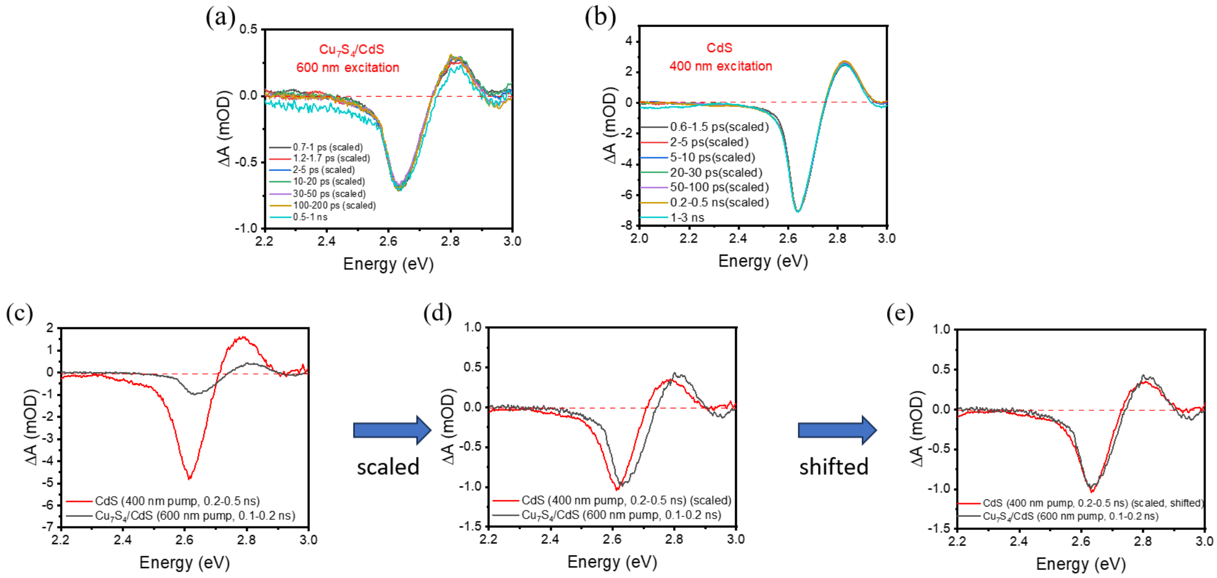


**Fig. S14**. (a) Scaled TA Spectra for $Cu_7S_4$/CdS with 600 nm Excitation. (b) Scaled TA Spectra for CdS with 400 nm Excitation. Spectra at different delay times overlap well after scaling. (c-e)After scaling, the spectra for $Cu_7S_4$/CdS and bare CdS collected are in good agreement.

**Figure S15. Spectra for bare $Cu_7S_4$ NCs and CdS NRs (600 nm excitation).**

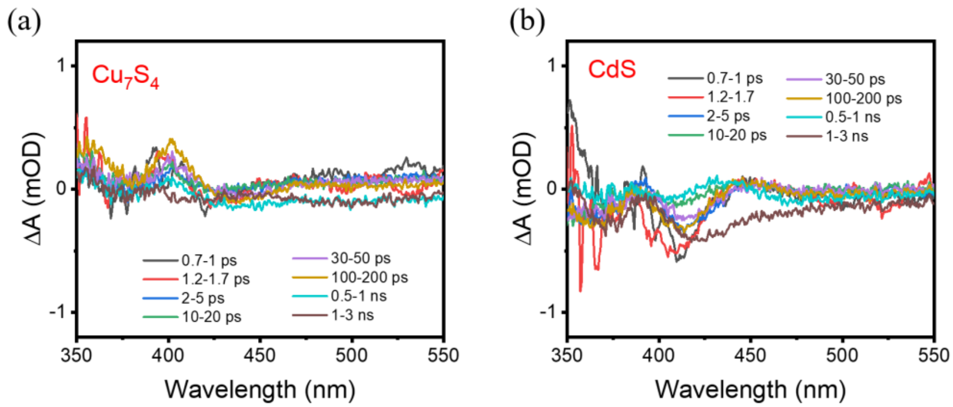


**Fig. S15** After 600 nm photoexcitation TAs of: (a) $Cu_7S_4$ NCs and (b) CdS NRs. Note: $Cu_7S_4$/CdS NRs were tested under the same conditions (Fig. 6a).

**Figure S16. Spectra for $Cu_7S_4$, CdS, and $Cu_7S_4$/CdS (800 nm excitation).**

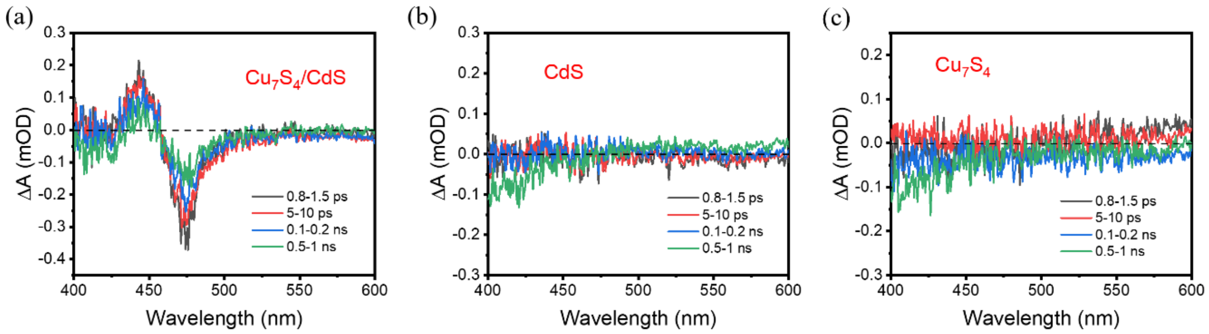


**Fig. S16** After 800 nm photoexcitation TAs of: (a) $Cu_7S_4$/CdS NRs, (b) CdS NRs, and (c) $Cu_7S_4$ NCs.

**Figure S17. Fluence-dependence for XB in $Cu_7S_4$/CdS (600 nm excitation).**

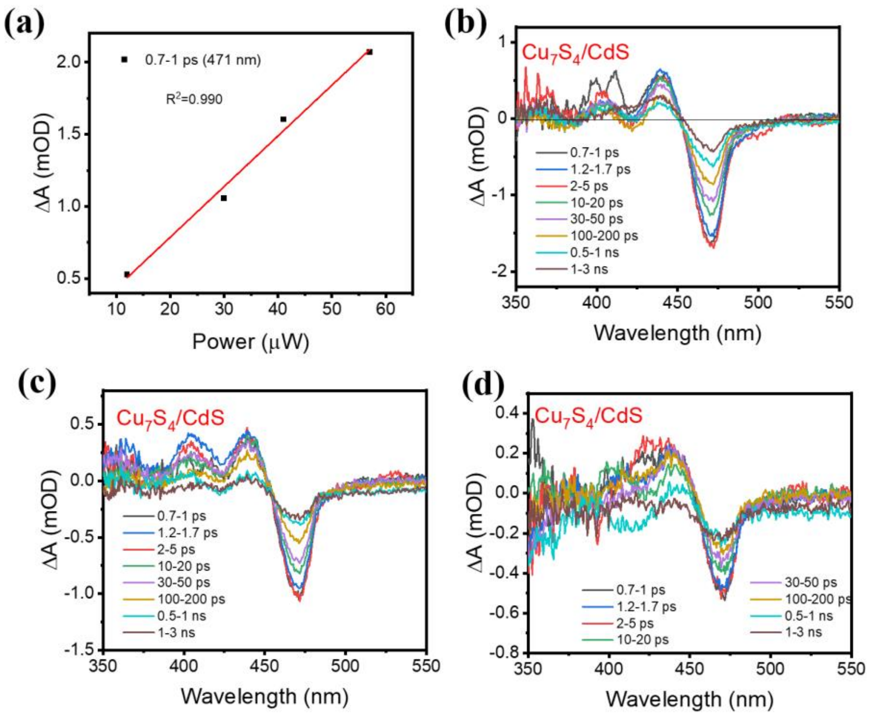


**Fig. S17** (a) Fluence-dependence for the DICTT induced XB in $Cu_7S_4$/CdS NRs. (b) Spectra at 41 $\mu$W. (c) Spectra at 30 $\mu$W. (d) Spectra at 12 $\mu$W.

## Reference

(1) Liu, Y.; Yang, W.; Chen, Q.; Cullen, D. A.; Xie, Z.; Lian, T. Pt Particle Size Affects Both the Charge Separation and Water Reduction Efficiencies of CdS–Pt Nanorod Photocatalysts for Light Driven H2 Generation. *Journal of the American Chemical Society* **2022**. DOI: 10.1021/jacs.1c11745.

(2) Cui, J.; Jiang, R.; Xu, S.; Hu, G.; Wang, L. Cu7 S4 Nanosuperlattices with Greatly Enhanced Photothermal Efficiency. *Small* **2015**, *11* (33), 4183-4190. DOI: 10.1002/smll.201500845.

(3) Wu, K.; Chen, J.; McBride, J. R.; Lian, T. Efficient hot-electron transfer by a plasmon-induced interfacial charge-transfer transition. *science* **2015**, *349* (6248), 632-635.

(4) Zhu, H.; Yang, Y.; Hyeon-Deuk, K.; Califano, M.; Song, N.; Wang, Y.; Zhang, W.; Prezhdo, O. V.; Lian, T. Auger-assisted electron transfer from photoexcited semiconductor quantum dots. *Nano Lett* **2014**, *14* (3), 1263-1269. DOI: 10.1021/nl4041687.

(5) Brus, L. E. Electron–electron and electron‐hole interactions in small semiconductor crystallites: The size dependence of the lowest excited electronic state. *The Journal of Chemical Physics* **1984**, *80* (9), 4403-4409. DOI: 10.1063/1.447218.

(6) Cui, J.; Li, Y.; Liu, L.; Chen, L.; Xu, J.; Ma, J.; Fang, G.; Zhu, E.; Wu, H.; Zhao, L.; et al. Near-Infrared Plasmonic-Enhanced Solar Energy Harvest for Highly Efficient Photocatalytic Reactions. *Nano Lett* **2015**, *15* (10), 6295-6301. DOI: 10.1021/acs.nanolett.5b00950.

(7) Lian, Z.; Sakamoto, M.; Vequizo, J. J. M.; Ranasinghe, C. S. K.; Yamakata, A.; Nagai, T.; Kimoto, K.; Kobayashi, Y.; Tamai, N.; Teranishi, T. Plasmonic p-n Junction for Infrared Light to Chemical Energy Conversion. *J Am Chem Soc* **2019**, *141* (6), 2446-2450. DOI: 10.1021/jacs.8b11544.

(8) Lian, Z.; Wu, F.; Zhong, Y.; Zi, J.; Li, Z.; Wang, X.; Nakagawa, T.; Li, H.; Sakamoto, M. Tuning plasmonic p–n junction for efficient infrared-light-responsive hydrogen evolution. *Applied Catalysis B: Environmental* **2022**, *318*. DOI: 10.1016/j.apcatb.2022.121860.

(9) Yang, W.; Liu, Y.; McBride, J. R.; Lian, T. Ultrafast and Long-Lived Transient Heating of Surface Adsorbates on Plasmonic Semiconductor Nanocrystals. *Nano Lett* **2021**, *21* (1), 453-461. DOI: 10.1021/acs.nanolett.0c03911.
